# Supplementary material for: Assessing eligibility for lung cancer screening using parsimonious ensemble machine learning models: A development and validation study
Source: PLoS Med. 2023 Oct 3;20(10):e1004287. doi: 10.1371/journal.pmed.1004287 (PMC10547178; doi:10.1371/journal.pmed.1004287)
Supplement: S1 Appendix — (PDF) [file pmed.1004287.s001.pdf]

Supplementary Material: Assessing eligibility for lung cancer screening using parsimonious machine learning models: A development and validation study

Contents

Participant flow diagrams **4**

Fig A: Flow diagram of UK Biobank participants 4

Fig B: Flow diagram of National Lung Screening Trial (NLST) participants 4

Fig C: Flow diagram of Prostate, Lung, Colorectal, and Ovarian Cancer Screening (PLCO) Trial participants 5

Variable recoding, missing data, and multiple imputation **6**

UK Biobank: Recoding smoking variables 6

UK Biobank: Missing data & multiple imputation 6

Table A: Distribution of complete data across smoking variables in our development cohort (UK Biobank) 6

Fig D: Nelson-Aalen curves of probability of (a) developing and (b) dying from lung cancer amongst ever-smokers with different patterns of missing smoking data in the UK Biobank. Shaded areas refer to 95% confidence intervals surrounding each Nelson-Aalen curve. 7

Fig E: Nelson-Aalen curves of probability of developing (a) and dying from (b) lung cancer amongst non-occasional ever-smokers not missing smoking intensity and duration in the UK Biobank. Shaded areas refer to 95% confidence intervals surrounding each Nelson-Aalen curve. 7

Fig F: Nelson-Aalen curves of probability of developing (a) and dying from (b) lung cancer amongst ever-smokers with smoking intensity and duration, ever-smokers missing smoking intensity and duration, and never-smokers in the UK Biobank. Shaded areas refer to 95% confidence intervals surrounding each Nelson-Aalen curve. 8

UK Biobank Imputation 8

NLST & PLCO: Missing data and multiple imputation 8

Model development **9**

Variable selection 9

Table B: Candidate variables 10

Fig G: The contribution of individual variables to predictions in UCLFull-D (predicting risk of lung cancer death). 10

Table C: Predictors used in comparator prognostic models 11

Cox models 12

Variable importance and interactions **12**

Supplementary Results **13**

Table D: Descriptive characteristics of UK Biobank ever-smokers by outcome 14

Table E: Descriptive characteristics of National Lung Screening Trial (NLST) control ever-smokers by outcome 16

Table F: Descriptive characteristics of Prostate, Lung, Colorectal, and Ovarian Cancer Screening (PLCO) Trial radiography arm ever-smokers by outcome 18

Table G: Descriptive characteristics of all Prostate, Lung, Colorectal, and Ovarian Cancer Screening (PLCO) Trial ever-smokers by outcome 20
Table H: Outcomes by dataset 22
Details of UCL-D 23
Fig H: The UCL-D ensemble and constituent pipelines (predicted outcome is five-year risk of death from lung cancer). 23
Table I: Hyperparameters for the AdaBoost and LightGBM machine learning algorithms in UCL-D 23
Details of UCL-I 23
Fig I: Details of the UCL-I ensemble (predicted outcome is five-year risk of developing lung cancer). 24
Table J: Hyperparameters for the AdaBoost, LightGBM, and CatBoost machine learning algorithms in UCL-I 24
Table K: Discrimination (AUC) of models amongst ever-smokers in the UK Biobank 25
Table L: Brier scores in the PLCO chest radiography arm 26
Table M: Brier scores of models amongst ever-smokers in the UK Biobank 27
Table N: Calibration of models amongst ever-smokers in the UK Biobank 28
Table O: Model sensitivity and sensitivity at specified risk thresholds in the PLCO dataset 29
Fig J: Outcomes by eligibility for either UCL-D or UCL-I, but not both UCL models 30
Fig K: Net benefit of models in the UK Biobank 31
Full Models 33
UCLFull-D 33
UCLFull-I 33
Table P: Discriminative accuracy (AUC) in the whole PLCO cohort 34
Table Q: Overall performance (Brier scores) in the whole PLCO cohort 36
Fig L: Calibration curves for UCL models in the whole PLCO cohort 38
Fig M: Net benefit of UCL models in the whole Prostate, Lung, Colorectal, and Ovarian Cancer Screening (PLCO) Trial cohort 39
Table R: Sensitivity and sensitivity of UCLFull-D and UCLFull-I at specified risk thresholds in the PLCO dataset 40
References 41

Authors and affiliations:

Thomas Callender[1], Fergus Imrie[2], Bogdan Cebere[3], Nora Pashayan[4], Neal Navani[1], Mihaela van der Schaar[3,5,6*], Sam M Janes[1*]

[1] Department of Respiratory Medicine, 5 University Street, University College London
[2] Department of Electrical and Computer Engineering, University of California, Los Angeles
[3] Department of Applied Mathematics and Theoretical Physics, University of Cambridge
[4] Department of Applied Health Research, 1-19 Torrington Place, University College London

[5] Cambridge Centre for AI in Medicine, University of Cambridge
[6] Alan Turing Institute

*Joint senior authors

Participant flow diagrams

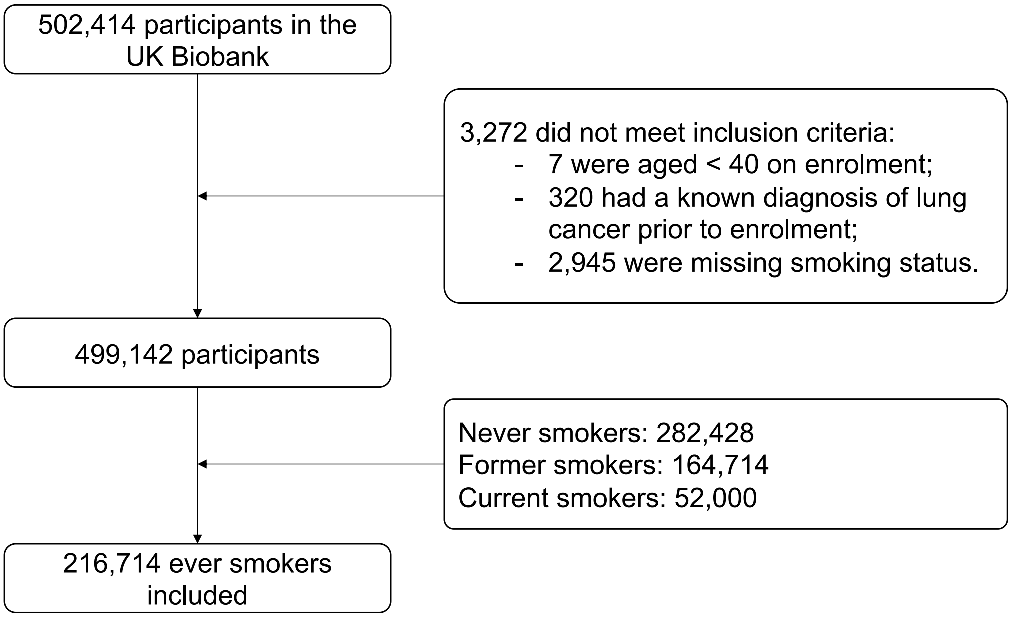

Fig A: Flow diagram of UK Biobank participants

From the UK Biobank, we included 216,714 ever-smoking individuals without a known diagnosis of lung cancer (ICD-10 codes C33-C34) aged 40 or more at baseline (Fig A).

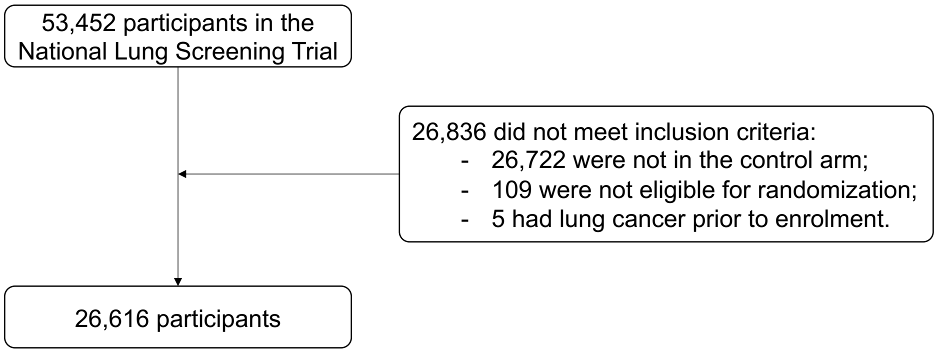

Fig B: Flow diagram of National Lung Screening Trial (NLST) participants

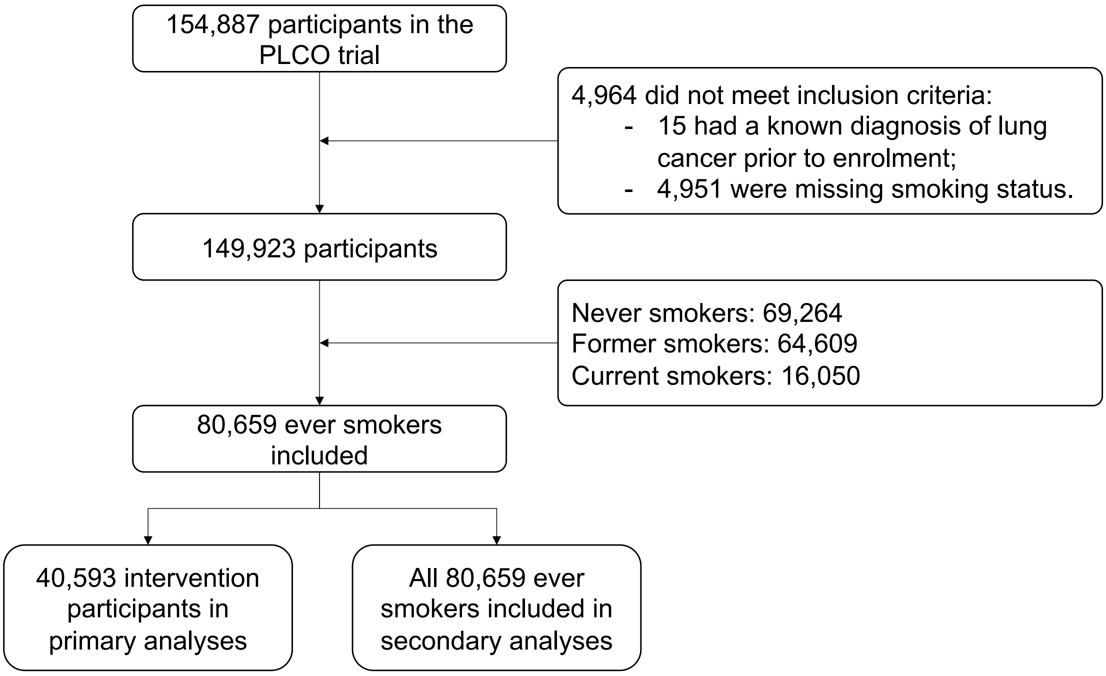

Fig C: Flow diagram of Prostate, Lung, Colorectal, and Ovarian Cancer Screening (PLCO) Trial participants

Variable recoding, missing data, and multiple imputation

UK Biobank: Recoding smoking variables

To determine smoking status in the UK Biobank, we used their self-recorded smoking status (field 20116). We then re-coded the 9,010 participants who had declared (field 1249) they had never tried smoking, had smoked only once or twice, or less than 100 cigarettes in their lifetime (field 2644) to non-smokers. The final numbers by smoking status were: 282,428 never smokers, 165,714 former smokers and 52,000 current smokers.

UK Biobank: Missing data & multiple imputation

As the most influential risk factor for lung cancer, we specifically analysed patterns of missingness in smoking variables. Three smoking variables were key: age start smoking, age stop smoking, and smoking intensity (number of cigarettes smoked per day). Based on these three variables, variables such as smoking duration and pack-years are calculated.

In the UK Biobank development cohort, just over two-thirds of all participants (68.9%), and over five-sixths of those who developed lung cancer (86.3%) and of those who died from lung cancer (86.8%) during follow-up had complete data across all included predictors (Table A). However, we noted that missing all three smoking variables was the most common pattern amongst all participants, occurring in nearly one-quarter of ever-smokers in our development dataset (22.6%).

Table A: Distribution of complete data across smoking variables in our development cohort (UK Biobank)

Variables missing	All participants, n=216,714 [n, (%)]	Developed lung cancer, n=3,449 [n, (%)]	Died from lung cancer, n=2,137 [n, (%)]
Complete data	149,328 (68.91)	2,977 (86.31)	1,854 (86.76)
1	10,264 (4.74)	228 (6.61)	137 (6.41)
2	8,074 (3.73)	56 (1.62)	35 (1.64)
3	49,048 (22.63)	188 (5.45)	111 (5.19)

Within the context of prediction modelling, our interest is the relationship between missingness in a variable and the outcome – the informativeness of missingness – and the impact this has on predictive performance [1]. Amongst those with an outcome of interest, the most common pattern was to be missing one variable; missing one variable was not associated with a higher cumulative risk of developing lung cancer (log-rank test $p = 0.07$) or dying from lung cancer (log-rank test $p = 0.31$). By contrast, as shown in Fig D, missing three variables - the next most common pattern seen amongst those with an outcome of interest - was associated with a different risk of an outcome relative to those with complete data (log-rank test $p < 0.001$).

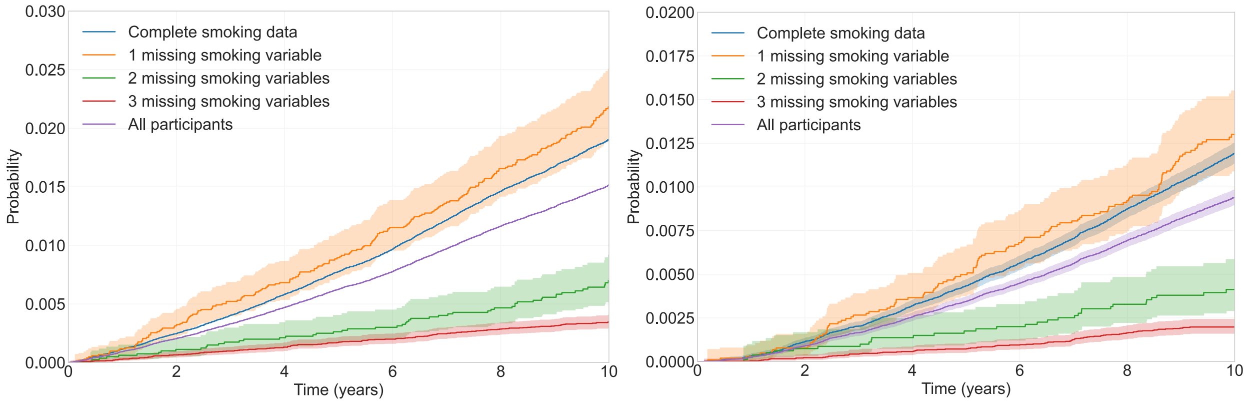

Fig D: Nelson-Aalen curves of probability of (a) developing and (b) dying from lung cancer amongst ever-smokers with different patterns of missing smoking data in the UK Biobank. Shaded areas refer to 95% confidence intervals surrounding each Nelson-Aalen curve.

Reviewing those participants who were missing all three key smoking variables, nearly all (99.9%, n=49,030) were recorded as former smokers who had only smoked occasionally (UK Biobank fields 20116 & 1249). These individuals were not questioned about the number of cigarettes they smoked per day. On removing these participants from analysis, the relationship between missingness and the outcomes of interest inverted ($p < 0.001$ for risk of developing or dying from lung cancer, Fig E).

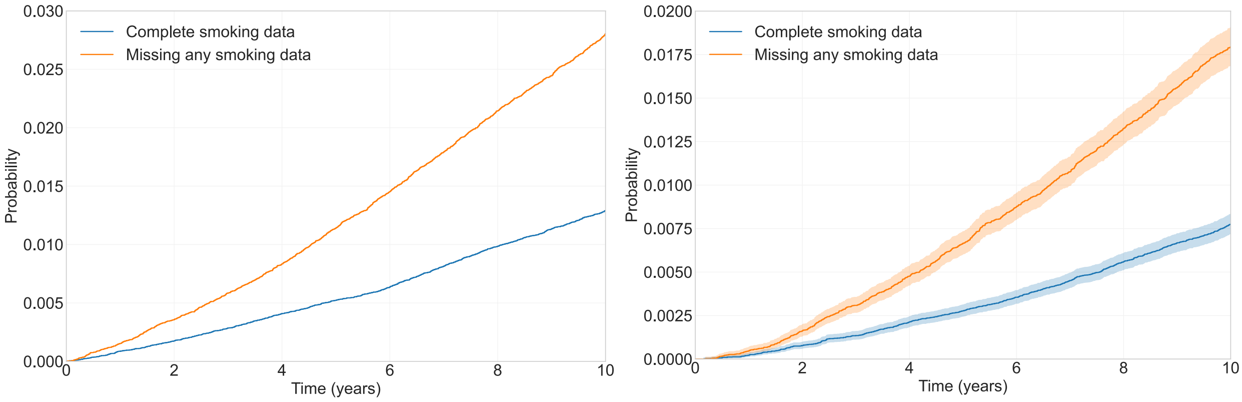

Fig E: Nelson-Aalen curves of probability of developing (a) and dying from (b) lung cancer amongst non-occasional ever-smokers not missing smoking intensity and duration in the UK Biobank. Shaded areas refer to 95% confidence intervals surrounding each Nelson-Aalen curve.

However, the former occasional smokers themselves showed different outcome profiles to both current smokers recorded to only smoke occasionally and non-smokers (Fig F). We therefore included them in our analyses.

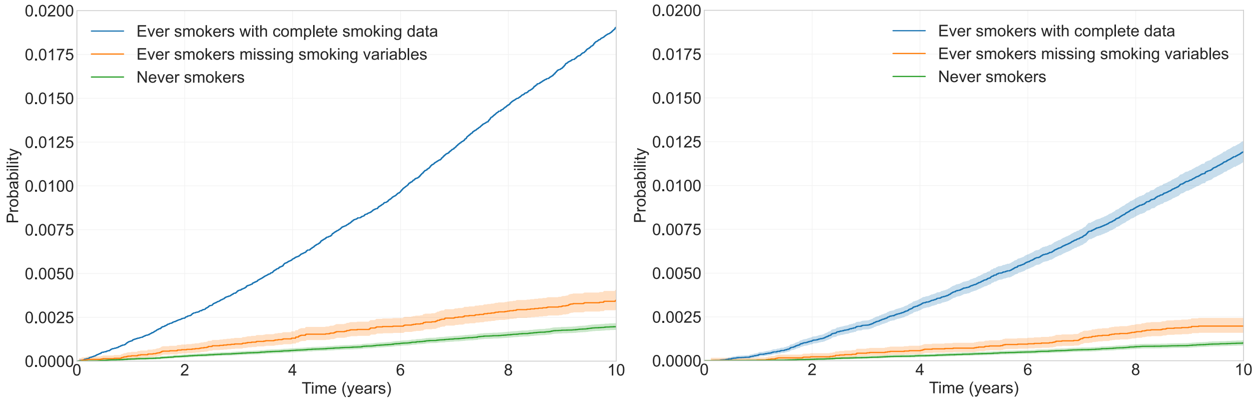

Fig F: Nelson-Aalen curves of probability of developing (a) and dying from (b) lung cancer amongst ever-smokers with smoking intensity and duration, ever-smokers missing smoking intensity and duration, and never-smokers in the UK Biobank. Shaded areas refer to 95% confidence intervals surrounding each Nelson-Aalen curve.

UK Biobank Imputation

We generated 10 imputed datasets using multiple imputation by chained equations (MICE) and a tree-based Gradient Boosting imputation model (LightGBM) to avoid assumptions about statistical relationships between the variables, implemented with the Python package miceforest [2]. For each candidate predictor with missing data, a model was fit that consisted of both a common pool of candidate variables and lung cancer outcomes as well as a bespoke set of predictors that were specifically correlated with missingness in the variable of interest [3].

To account for the systematic difference between former ‘occasional’ smokers missing cigarettes smoked per day and others, we imputed this number by taking a random draw from a *Gamma*(1.5626, 6.4) distribution. This equates to a median of 8 cigarettes smoked per *month* (interquartile range: 4-14, 2.5th and 97.5th centile: 0.8 and 31). We repeated this for each of the 10 imputed datasets.

Models were developed using a single imputed dataset as there are no established methods for pooling machine learning model hyperparameters between imputed datasets. However, to assess model performance, we pooled all ten imputed datasets using Rubin’s rules.

NLST & PLCO: Missing data and multiple imputation

Overall missingness was <5% for all relevant variables in both the National Lung Screening Trial (NLST) and Prostate, Lung, Colorectal, and Ovarian Cancer Screening (PLCO) Trial datasets. Given the low level of missingness, we generated five imputed NLST and PLCO datasets. In both cases we used multiple imputation by chained equations with predictive mean matching, implemented as described above. Note there was no missingness amongst age, smoking intensity, and smoking duration variables in the NLST trial.

Model development

We fit models using AutoPrognosis [4,5], and for comparison and model validation with Cox proportional hazards regression.

In this analysis, AutoPrognosis searched for optimal pipelines – where each pipeline consists of three stages: dimensionality reduction, predictor pre-processing, and the model algorithm – from 252 potential combinations. The following algorithms were considered:

- Dimensionality reduction: none, variance thresholding, principal component analysis, independent component analysis.
- Predictor pre-processing: none, normalisation, polynomial interactions between features, scaling each predictor using its maximum absolute value, min-max scaling, uniform transformation, standardisation.
- Modelling algorithms: logistic regression, linear discriminant analysis, quadratic discriminant analysis, bagging, random forests, Adaboost [6–8], CatBoost [9,10], LightGBM [11,12], XGBoost [13,14].

AutoPrognosis uses Bayesian optimisation for pipeline selection [4], whilst the hyperparameters of each modelling algorithm trialled were tuned using Optuna [15]. Ensembles are generated using both stacking and aggregating methods from the Python package combo [16] and by Bayesian model averaging [4]. All pipelines and ensembles were trained in the entire development dataset. Five-fold cross-validation was used to evaluate the performance of each pipeline and each ensemble of pipelines, and the highest performing ensemble selected. We considered ensembles that consisted of up to four different modelling pipelines. In other words, should a single pipeline, for example one involving no dimensionality reduction, predictor standardisation, and subsequently the machine learning algorithm LightGBM, have had greater discrimination than an ensemble of several pipelines, this would have been selected. The maximum number of pipelines was set pragmatically as a balance between model performance and the computational time required for model training.

Variable selection

We considered candidate variables based on evidence of a causal association with lung cancer and/or presence in another risk prediction model [17]. Candidate variables (Table B) needed to be present in all three of the UK Biobank, NLST and PLCO datasets.

We used predictive performance as determined with the AUC in our development dataset to derive the predictors used in our full models (presented in more detail in a sub-section below). On review of the feature importance in our full model (Fig G), three variables: age, smoking duration, and pack-years were found to be driving most predictions, such that we then developed models using these three variables. As the difference in discrimination between the full models and the three-variable models was not statistically significant, we took forward the three-variable models. We present the full models below to show the limited benefits of including more variables when using the same machine learning framework.

**Table B:** Candidate variables
1. Age
2. Sex
3. Body mass index
4. Weight
5. Ethnicity (White, Black, Asian, Other)
6. Highest qualification (degree, some college, post-secondary school, secondary school, none of the above)
7. Smoking status
8. Age started smoking
9. Age stopped smoking
10. Smoking duration
11. Quit-years
12. Smoking intensity (cigarettes per day)
13. Pack-years
14. COPD
15. Number of previous cancers
16. Family history of cancer in father
17. Family history of cancer in mother
18. Family history of cancer in sibling(s)
19. Family history of cancer (total number)

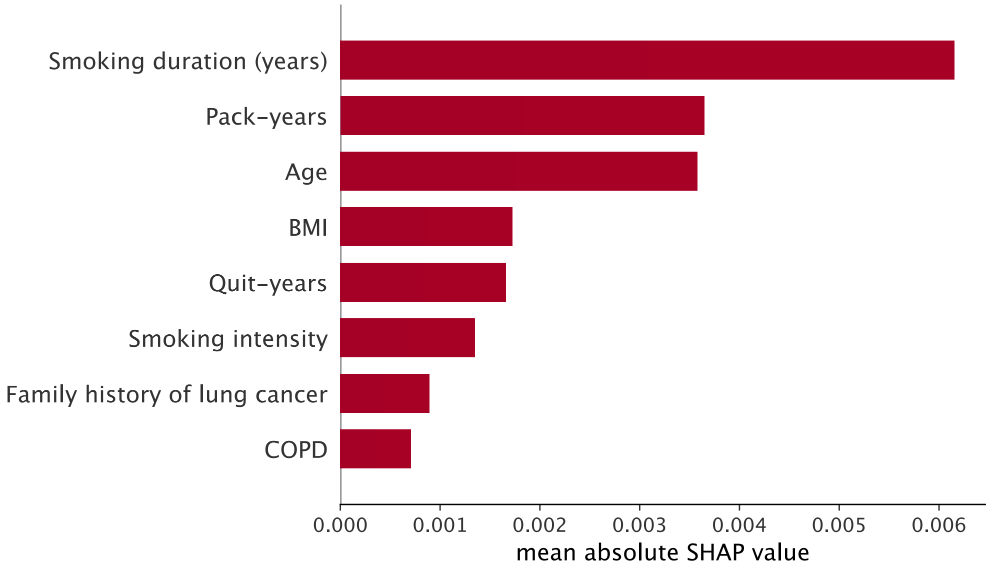

Fig G: The contribution of individual variables to predictions in UCLFull-D (predicting risk of lung cancer death).

Further details of UCLFull-D are presented in a sub-section of S1 Appendix below. Shapely Additive Explanation (SHAP) values describe the mean contribution made by each predictor to overall predictions (see section Variable Importance and Interaction for further details).

Table C: Predictors used in comparator prognostic models

Model	Predictors
UCL-D & UCL-I	1. Age<br>2. Smoking duration (years)<br>3. Pack-years[a]
PLCOm2012 [18]	1. Age<br>2. Smoking status<br>3. Smoking duration (years)<br>4. Smoking intensity (number of cigarettes per day)<br>5. Quit-years (if former smoker)<br>6. Ethnicity (White, Black, Hispanic, Asian, American Indian or Alaskan Native, Native Hawaiian or Pacific Islander)<br>7. Highest qualification<br>8. Body-mass index<br>9. Chronic obstructive pulmonary disease (COPD)<br>10. Personal history of cancer<br>11. Family history of lung cancer
LCRAT & LCDRAT [19]	1. Age<br>2. Sex<br>3. Ethnicity (White non-hispanic, Black non-Hispanic, Hispanic, Asian or other)<br>4. Highest qualification<br>5. Body-mass index<br>6. Smoking duration (years)<br>7. Smoking intensity (number of cigarettes per day)<br>8. Quit-years (if former smoker)<br>9. Pack-years<br>10. History of emphysema<br>11. Family history of lung cancer
LLP versions 2 & 3 [20]	1. Age<br>2. Sex<br>3. Smoking duration (years)<br>4. Pneumonia,<br>5. Asthma,<br>6. Bronchitis, emphysema, or COPD<br>7. Tuberculosis<br>8. Asbestos exposure<br>9. Personal history of cancer<br>10. Family history of lung cancer (none, before age 60, after age 70)

[a]Pack-years combine smoking duration with the number of cigarettes smoked, with one pack-year equivalent to smoking 20 cigarettes per day for a year.
Abbreviations: LLP, Liverpool Lung Project; UCL-D predicts lung cancer death; UCL-I predicts occurrence of lung cancer; LCDRAT, Lung Cancer Death Risk Assessment Tool; LCRAT, Lung Cancer Risk Assessment Tool; COPD, chronic obstructive pulmonary disease.

Cox models

We developed Cox models using the survival [21] and rms [22] packages in R. To account for non-linear relationships between age, smoking duration, pack-years and lung cancer (death), we used restricted cubic splines modelled with three knots. In common with both Tammemägi [18], Katki [19], and their colleagues, we found that no interactions between the three variables were significant within a Cox framework. Further, Cox models without including interaction terms had lower Akaike Information Criterion (AIC) values. We present the c-index for models both with and without interactions in the Appendix results.

Variable importance and interactions

We used Kernel Shapely Additive Explanations (SHAP) to disentangle the contribution of different variables to predictions, including interactions between variables.[23] Kernel SHAP is a feature attribution method designed to explain model predictions through the calculation of Shapely values, whose values represent the contribution made by individual predictors to the overall prediction [23].

Shapely values are calculated by iteratively passing each predictor through a model and attributing changes in the predicted outcomes for an individual in its absence to the predictor. As an example, for an individual with an age of 55, smoking duration of 30 years and pack-years of 60, the change in prediction made if the model only used smoking duration and pack-years can be attributed to age. These attributions are subsequently averaged across the dataset to get the final Shapely values for each predictor [24]. With SHAP, the result is a linear model $\phi_0 + \sum \phi_i x_i$, where ϕ_0 is an intercept corresponding to the average predicted risk in the dataset being explained, along with the SHAP values ϕ_i for each of the predictors x_i (i.e., age, smoking duration, and pack-years) [23].

Supplementary Results

Table D: Descriptive characteristics of UK Biobank ever-smokers by outcome

	Lung cancer n=3,449	No lung cancer n=213,265	Lung cancer death n=2,137	No lung cancer death n=214,577	All participants n=216,714
Age [n, (%)]					
<50	123 (3.57)	43,047 (20.18)	75 (3.51)	43,095 (20.08)	43,170 (19.92)
50-54	243 (7.05)	29,834 (13.99)	152 (7.11)	29,925 (13.95)	30,077 (13.88)
55-59	556 (16.12)	38,983 (18.28)	347 (16.24)	39,192 (18.26)	39,539 (18.24)
60-64	1,180 (34.21)	56,115 (26.31)	712 (33.32)	56,583 (26.37)	57,295 (26.44)
65-69	1,316 (38.16)	44,204 (20.73)	834 (39.03)	44,686 (20.83)	45,520 (21.0)
≥70	31 (0.9)	1,082 (0.51)	17 (0.8)	1,096 (0.51)	1,113 (0.51)
Missing	0 (0.0)	0 (0.0)	0 (0.0)	0 (0.0)	0 (0.0)
Sex – Female [n, (%)]	1,574 (45.64)	102,124 (47.89)	936 (43.8)	102,762 (47.89)	103,698 (47.85)
Missing	0 (0.0)	0 (0.0)	0 (0.0)	0 (0.0)	0 (0.0)
Ethnicity – White [n, (%)]	3,368 (98.02)	204,887 (96.44)	2,097 (98.36)	206,158 (96.45)	208,255 (96.47)
Missing	13 (0.38)	817 (0.38)	5 (0.23)	825 (0.38)	830 (0.38)
Highest qualification [n, (%)]					
Degree	478 (14.3)	59,227 (28.29)	276 (13.29)	59,429 (28.22)	59,705 (28.07)
Some college	310 (9.28)	16,191 (7.73)	200 (9.63)	16,301 (7.74)	16,501 (7.76)
Post-secondary school	450 (13.46)	33,138 (15.83)	275 (13.24)	33,313 (15.82)	33,588 (15.79)
Secondary school	725 (21.69)	56,921 (27.19)	437 (21.04)	57,209 (27.17)	57,646 (27.11)
None of the above	1,379 (41.26)	43,852 (20.95)	889 (42.8)	44,342 (21.06)	45,231 (21.27)
Missing	107 (3.1)	3936 (1.85)	60 (2.81)	3983 (1.86)	4043 (1.87)
In paid employment/self-employed [n, (%)]	1,071 (31.16)	115,762 (54.47)	629 (29.49)	116,204 (54.35)	116,833 (54.1)
Missing	12 (0.35)	755 (0.35)	4 (0.19)	763 (0.36)	767 (0.35)
Body mass index					
<18.5	46 (1.35)	1,038 (0.49)	29 (1.37)	1,055 (0.49)	1,084 (0.5)
18.5-24	1,046 (30.65)	61,669 (29.08)	652 (30.78)	62,063 (29.09)	62,715 (29.1)
25-29	1,437 (42.1)	92,835 (43.77)	904 (42.68)	93,368 (43.76)	94,272 (43.75)
30-34	665 (19.48)	40,804 (19.24)	403 (19.03)	41,066 (19.25)	41,469 (19.24)
≥35	219 (6.42)	15,735 (7.42)	130 (6.14)	15,824 (7.42)	15,954 (7.4)
Missing	36 (1.04)	1,184 (0.56)	19 (0.89)	1,201 (0.56)	1,220 (0.56)
Household income (GBP £)					
<18,000	1,318 (47.6)	47,749 (26.13)	864 (50.2)	48,203 (26.23)	49,067 (26.45)
18,000-30,999	790 (28.53)	48,233 (26.39)	492 (28.59)	48,531 (26.4)	49,023 (26.42)
31,000-51,999	404 (14.59)	45,716 (25.01)	230 (13.36)	45,890 (24.97)	46,120 (24.86)
52,000-100,000	208 (7.51)	32,812 (17.95)	110 (6.39)	32,910 (17.9)	33,020 (17.8)
>100,000	49 (1.77)	8,247 (4.51)	25 (1.45)	8,271 (4.5)	8,296 (4.47)
Missing	680 (19.72)	30,508 (14.31)	416 (19.47)	30,772 (14.34)	31,188 (14.39)

Continued…

Smoking status					
Former	1,817 (52.68)	162,897 (76.38)	1,073 (50.21)	163,641 (76.26)	164,714 (76.01)
Current	1,632 (47.32)	50,368 (23.62)	1,064 (49.79)	50,936 (23.74)	52,000 (23.99)
Missing	0 (0.0)	0 (0.0)	0 (0.0)	0 (0.0)	0 (0.0)
Age started smoking					
<16	1,223 (38.34)	47,210 (30.34)	792 (39.94)	47,641 (30.38)	48,433 (30.5)
16-20	1,658 (51.97)	87,974 (56.54)	997 (50.28)	88,635 (56.53)	89,632 (56.45)
>20	309 (9.69)	20,403 (13.11)	194 (9.78)	20,518 (13.09)	20,712 (13.04)
Missing	259 (7.51)	57,678 (27.05)	154 (7.21)	57,783 (26.93)	57,937 (26.73)
Years smoked					
<10	54 (1.7)	16,910 (10.9)	31 (1.57)	16,933 (10.83)	16,964 (10.71)
10-19	176 (5.53)	36,641 (23.62)	105 (5.31)	36,712 (23.48)	36,817 (23.25)
20-29	386 (12.14)	38,039 (24.52)	201 (10.16)	38,224 (24.45)	38,425 (24.27)
30-39	787 (24.75)	35,610 (22.95)	480 (24.27)	35,917 (22.97)	36,397 (22.99)
≥40	1,777 (55.88)	27,954 (18.02)	1,161 (58.7)	28,570 (18.27)	29,731 (18.78)
Missing	269 (7.8)	58,111 (27.25)	159 (7.44)	58,221 (27.13)	58,380 (26.94)
Cigarettes per day (median, IQR)					
1-10	547 (18.26)	41,797 (28.4)	338 (18.14)	42,006 (28.32)	42,344 (28.2)
11-20	1,575 (52.57)	76,880 (52.24)	977 (52.44)	77,478 (52.24)	78,455 (52.24)
21-30	545 (18.19)	18,653 (12.67)	336 (18.04)	18,862 (12.72)	19,198 (12.78)
31-40	240 (8.01)	6,837 (4.65)	153 (8.21)	6,924 (4.67)	7,077 (4.71)
>40	89 (2.97)	3,013 (2.05)	59 (3.17)	3,043 (2.05)	3,102 (2.07)
Missing	453 (13.13)	66,085 (30.99)	274 (12.82)	66,264 (30.88)	66,538 (30.7)
Pack-years of smoking [n, (%)]					
<10	155 (5.21)	35,067 (23.96)	91 (4.91)	35,131 (23.82)	35,222 (23.59)
10-19	371 (12.46)	39,543 (27.02)	218 (11.76)	39,696 (26.92)	39,914 (26.73)
20-29	538 (18.07)	28,933 (19.77)	321 (17.31)	29,150 (19.77)	29,471 (19.74)
30-39	595 (19.99)	20,001 (13.67)	362 (19.53)	20,234 (13.72)	20,596 (13.79)
≥40	1,318 (44.27)	22,807 (15.58)	862 (46.49)	23,263 (15.77)	24,125 (16.16)
Missing	472 (13.69)	66,914 (31.38)	283 (13.24)	67,103 (31.27)	67,386 (31.09)
Personal history of cancer [n, (%)]	515 (14.93)	18,871 (8.85)	291 (13.62)	19,095 (8.9)	19,386 (8.95)
Missing	0 (0.0)	0 (0.0)	0 (0.0)	0 (0.0)	0 (0.0)
Chronic Obstructive Pulmonary Disease (COPD) / Emphysema / Bronchitis [n, (%)]	409 (11.9)	6,207 (2.92)	248 (11.64)	6,368 (2.97)	6,616 (3.06)
Missing	13 (0.38)	441 (0.21)	7 (0.33)	447 (0.21)	454 (0.21)
Family history of lung cancer [n, (%)]	797 (23.72)	27,968 (13.36)	473 (22.78)	28,292 (13.43)	28,765 (13.52)
Missing	89 (2.58)	3,855 (1.81)	61 (2.85)	3,883 (1.81)	3,944 (1.82)

Table E: Descriptive characteristics of National Lung Screening Trial (NLST) control ever-smokers by outcome

	Lung cancer n=960	No lung cancer n=25,656	Lung cancer death n=545	No lung cancer death n=26,071	All participants n=26,616
Age [n, (%)]					
55-59	247 (25.73)	11,137 (43.41)	139 (25.5)	11,245 (43.13)	11,384 (42.77)
60-64	299 (31.15)	7,871 (30.68)	165 (30.28)	8,005 (30.7)	8,170 (30.7)
65-69	250 (26.04)	4,491 (17.5)	141 (25.87)	4,600 (17.64)	4,741 (17.81)
70-74	164 (17.08)	2,157 (8.41)	100 (18.35)	2,221 (8.52)	2,321 (8.72)
Missing	0 (0.0)	0 (0.0)	0 (0.0)	0 (0.0)	0 (0.0)
Sex – Female [n, (%)]	390 (40.62)	10,529 (41.04)	212 (38.9)	10,707 (41.07)	10,919 (41.02)
Missing	0 (0.0)	0 (0.0)	0 (0.0)	0 (0.0)	0 (0.0)
Ethnicity – White [n, (%)]	871 (91.2)	23,294 (91.51)	492 (91.28)	23,673 (91.5)	24,165 (91.5)
Missing	5 (0.52)	201 (0.78)	6 (1.1)	200 (0.77)	206 (0.77)
Qualifications [n, (%)]					
Degree	214 (22.43)	7,999 (31.36)	116 (21.56)	8,097 (31.23)	8,213 (31.03)
Some college	209 (21.91)	5,863 (22.98)	121 (22.49)	5,951 (22.95)	6,072 (22.94)
Post-secondary school	430 (45.07)	9,670 (37.91)	247 (45.91)	9,853 (38.0)	10,100 (38.17)
Secondary school	67 (7.02)	1,144 (4.48)	40 (7.43)	1,171 (4.52)	1,211 (4.58)
None of the above	34 (3.56)	834 (3.27)	14 (2.6)	854 (3.29)	868 (3.28)
Missing	6 (0.62)	146 (0.57)	7 (1.28)	145 (0.56)	152 (0.57)
Body mass index [n, (%)]					
<18.5	13 (1.37)	227 (0.89)	10 (1.86)	230 (0.89)	240 (0.91)
18.5-24	339 (35.61)	6,963 (27.35)	193 (35.87)	7,109 (27.48)	7,302 (27.65)
25-29	400 (42.02)	11,042 (43.38)	221 (41.08)	11,221 (43.37)	11,442 (43.33)
30-34	144 (15.13)	5,075 (19.94)	88 (16.36)	5,131 (19.83)	5,219 (19.76)
≥35	56 (5.88)	2,149 (8.44)	26 (4.83)	2,179 (8.42)	2,205 (8.35)
Missing	8 (0.83)	200 (0.78)	7 (1.28)	201 (0.77)	208 (0.78)
Smoking status [n, (%)]					
Former	362 (37.71)	13,402 (52.24)	189 (34.68)	13,575 (52.07)	13,764 (51.71)
Current	598 (62.29)	12,254 (47.76)	356 (65.32)	12,496 (47.93)	12,852 (48.29)
Missing	0 (0.0)	0 (0.0)	0 (0.0)	0 (0.0)	0 (0.0)
Age started smoking [n, (%)]					
<16	408 (42.5)	9,524 (37.12)	229 (42.02)	9,703 (37.22)	9,932 (37.32)
16-20	471 (49.06)	13,304 (51.86)	268 (49.17)	13,507 (51.81)	13,775 (51.75)
>20	81 (8.44)	2,828 (11.02)	48 (8.81)	2,861 (10.97)	2,909 (10.93)
Missing	0 (0.0)	0 (0.0)	0 (0.0)	0 (0.0)	0 (0.0)
Years smoked [n, (%)]					
<10	0 (0.0)	0 (0.0)	0 (0.0)	0 (0.0)	0 (0.0)
10-19	1 (0.1)	66 (0.26)	0 (0.0)	67 (0.26)	67 (0.25)
20-29	14 (1.46)	1,749 (6.82)	10 (1.83)	1,753 (6.72)	1,763 (6.62)
30-39	196 (20.42)	10,296 (40.13)	110 (20.18)	10,382 (39.82)	10,492 (39.42)
≥40	749 (78.02)	13,545 (52.79)	425 (77.98)	13,869 (53.2)	14,294 (53.7)
Missing	0 (0.0)	0 (0.0)	0 (0.0)	0 (0.0)	0 (0.0)

Continued…

Cigarettes per day [n, (%)]					
<10	0 (0.0)	0 (0.0)	0 (0.0)	0 (0.0)	0 (0.0)
10-19	42 (4.38)	1,335 (5.2)	17 (3.12)	1,360 (5.22)	1,377 (5.17)
20-29	436 (45.42)	12,321 (48.02)	243 (44.59)	12,514 (48.0)	12,757 (47.93)
30-39	221 (23.02)	5,995 (23.37)	129 (23.67)	6,087 (23.35)	6,216 (23.35)
≥40	261 (27.19)	6,005 (23.41)	156 (28.62)	6,110 (23.44)	6,266 (23.54)
Missing	0 (0.0)	0 (0.0)	0 (0.0)	0 (0.0)	0 (0.0)
Pack-years of smoking [n, (%)]					
<10	0 (0.0)	0 (0.0)	0 (0.0)	0 (0.0)	0 (0.0)
10-19	0 (0.0)	0 (0.0)	0 (0.0)	0 (0.0)	0 (0.0)
20-29	0 (0.0)	4 (0.02)	0 (0.0)	4 (0.02)	4 (0.02)
30-39	112 (11.67)	6,753 (26.32)	59 (10.83)	6,806 (26.11)	6,865 (25.79)
≥40	848 (88.33)	18,899 (73.66)	486 (89.17)	19,261 (73.88)	19,747 (74.19)
Missing	0 (0.0)	0 (0.0)	0 (0.0)	0 (0.0)	0 (0.0)
Personal history of cancer [n, (%)]	59 (6.15)	1,138 (4.44)	29 (5.32)	1,168 (4.48)	1,197 (4.5)
Missing	0 (0.0)	0 (0.0)	0 (0.0)	0 (0.0)	0 (0.0)
Chronic Obstructive Pulmonary Disease (COPD) / Emphysema / Chronic bronchitis [n, (%)]	267 (27.81)	4,350 (16.96)	131 (24.04)	4,486 (17.21)	4,617 (17.35)
Missing	0 (0.0)	0 (0.0)	0 (0.0)	0 (0.0)	0 (0.0)
Family history of lung cancer [n, (%)]	256 (26.67)	5,478 (21.35)	142 (26.06)	5,592 (21.45)	5,734 (21.54)
Missing	0 (0.0)	0 (0.0)	0 (0.0)	0 (0.0)	0 (0.0)

Table F: Descriptive characteristics of Prostate, Lung, Colorectal, and Ovarian Cancer Screening (PLCO) Trial radiography arm ever-smokers by outcome

	Lung cancer n=1,734	No lung cancer n=38,859	Lung cancer death n=1,782	No lung cancer death n=38,811	All participants n=40,593
Age [n, (%)]					
55-59	357 (20.6)	13,608 (35.03)	382 (21.44)	13,583 (35.01)	13,965 (34.41)
60-64	514 (29.66)	12,109 (31.17)	541 (30.36)	12,082 (31.14)	12,623 (31.1)
65-69	548 (31.62)	8,569 (22.06)	560 (31.43)	8,557 (22.05)	9,117 (22.46)
≥70	314 (18.12)	4,565 (11.75)	299 (16.78)	4,580 (11.8)	4,879 (12.02)
Missing	1 (0.06)	8 (0.02)	0 (0.0)	9 (0.02)	9 (0.02)
Sex - Female [n, (%)]	641 (36.97)	16,251 (41.82)	630 (35.35)	16,262 (41.9)	16,892 (41.61)
Missing	0 (0.0)	0 (0.0)	0 (0.0)	0 (0.0)	0 (0.0)
Ethnicity - White [n, (%)]	1,520 (87.66)	34,298 (88.31)	1,535 (86.19)	34,283 (88.38)	35,818 (88.29)
Missing	0 (0.0)	23 (0.06)	1 (0.06)	22 (0.06)	23 (0.06)
Highest qualification [n, (%)]					
Degree	402 (23.2)	12,747 (32.85)	424 (23.79)	12,725 (32.84)	13,149 (32.44)
Some college	400 (23.08)	9,034 (23.28)	423 (23.74)	9,011 (23.25)	9,434 (23.27)
Post-secondary school	679 (39.18)	13,724 (35.37)	685 (38.44)	13,718 (35.4)	14,403 (35.53)
Secondary school	225 (12.98)	2,858 (7.37)	216 (12.12)	2,867 (7.4)	3,083 (7.61)
None of the above	27 (1.56)	437 (1.13)	34 (1.91)	430 (1.11)	464 (1.14)
Missing	1 (0.06)	59 (0.15)	0 (0.0)	60 (0.15)	60 (0.15)
Body mass index					
<18.5	18 (1.05)	292 (0.76)	22 (1.25)	288 (0.75)	310 (0.77)
18.5-24	660 (38.57)	12,083 (31.48)	656 (37.19)	12,087 (31.53)	12,743 (31.78)
25-29	708 (41.38)	16,572 (43.18)	739 (41.89)	16,541 (43.15)	17,280 (43.1)
30-34	263 (15.37)	6,772 (17.64)	269 (15.25)	6,766 (17.65)	7,035 (17.55)
≥35	62 (3.62)	2,664 (6.94)	78 (4.42)	2,648 (6.91)	2,726 (6.8)
Missing	23 (1.33)	476 (1.22)	18 (1.01)	481 (1.24)	499 (1.23)
Smoking status					
Current	772 (44.52)	7,301 (18.79)	818 (45.9)	7,255 (18.69)	8,073 (19.89)
Previous	962 (55.48)	31,558 (81.21)	964 (54.1)	31,556 (81.31)	32,520 (80.11)
Missing	0 (0.0)	0 (0.0)	0 (0.0)	0 (0.0)	0 (0.0)
Age started smoking					
<16	468 (27.29)	7,357 (19.04)	450 (25.57)	7,375 (19.11)	7,825 (19.39)
16-20	978 (57.03)	23,374 (60.49)	1,029 (58.47)	23,323 (60.43)	24,352 (60.35)
>20	269 (15.69)	7,907 (20.46)	281 (15.97)	7,895 (20.46)	8,176 (20.26)
Missing	19 (1.1)	221 (0.57)	22 (1.23)	218 (0.56)	240 (0.59)
Years smoked					
<10	23 (1.35)	4,688 (12.3)	17 (0.97)	4,694 (12.33)	4,711 (11.83)
1-19	98 (5.75)	7,788 (20.43)	98 (5.61)	7,788 (20.46)	7,886 (19.81)
20-29	184 (10.8)	8,064 (21.16)	206 (11.78)	8,042 (21.12)	8,248 (20.71)
30-39	416 (24.41)	9,068 (23.79)	418 (23.91)	9,066 (23.81)	9,484 (23.82)
≥40	983 (57.69)	8,505 (22.32)	1,009 (57.72)	8,479 (22.27)	9,488 (23.83)
Missing	30 (1.73)	746 (1.92)	34 (1.91)	742 (1.91)	776 (1.91)

Cigarettes per day [n, (%)]					
1-10	197 (11.41)	10,237 (26.39)	205 (11.54)	10,229 (26.41)	10,434 (25.76)
11-20	611 (35.38)	14,331 (36.95)	636 (35.79)	14,306 (36.93)	14,942 (36.88)
21-30	446 (25.83)	7,503 (19.35)	479 (26.96)	7,470 (19.29)	7,949 (19.62)
31-40	297 (17.2)	4,097 (10.56)	279 (15.7)	4,115 (10.62)	4,394 (10.85)
>40	176 (10.19)	2,616 (6.75)	178 (10.02)	2,614 (6.75)	2,792 (6.89)
Missing	7 (0.4)	75 (0.19)	5 (0.28)	77 (0.2)	82 (0.2)
Pack-years of smoking [n, (%)]					
<10	39 (2.3)	6,570 (17.26)	34 (1.95)	6,575 (17.3)	6,609 (16.63)
10-19	114 (6.71)	7,491 (19.69)	120 (6.88)	7,485 (19.69)	7,605 (19.13)
20-29	170 (10.01)	5,669 (14.9)	182 (10.44)	5,657 (14.88)	5,839 (14.69)
30-39	168 (9.89)	4,940 (12.98)	164 (9.4)	4,944 (13.01)	5,108 (12.85)
≥40	1,208 (71.1)	13,384 (35.17)	1,244 (71.33)	13,348 (35.12)	14,592 (36.71)
Missing	35 (2.02)	805 (2.07)	38 (2.13)	802 (2.07)	840 (2.07)
Personal history of cancer [n, (%)]	97 (5.59)	1,740 (4.48)	96 (5.39)	1,741 (4.49)	1,837 (4.53)
Missing	0 (0.0)	5 (0.01)	0 (0.0)	5 (0.01)	5 (0.01)
Chronic Obstructive Pulmonary Disease (COPD) / Emphysema / Bronchitis [n, (%)]	308 (17.76)	3,309 (8.52)	295 (16.55)	3,322 (8.56)	3,617 (8.91)
Missing	0 (0.0)	0 (0.0)	0 (0.0)	0 (0.0)	0 (0.0)
Family history of lung cancer [n, (%)]	292 (17.95)	4,274 (11.44)	297 (17.74)	4,269 (11.44)	4,566 (11.71)
Missing	107 (6.17)	1495 (3.85)	108 (6.06)	1494 (3.85)	1602 (3.95)

Table G: Descriptive characteristics of all Prostate, Lung, Colorectal, and Ovarian Cancer Screening (PLCO) Trial ever-smokers by outcome

	Lung cancer n=3,356	No lung cancer n=77,303	Lung cancer death n=3,534	No lung cancer death n=77,125	All participants n=80,659
Age [n, (%)]					
55-59	692 (20.63)	26,886 (34.79)	743 (21.02)	26,835 (34.8)	27,578 (34.2)
60-64	1,013 (30.19)	24,095 (31.18)	1,097 (31.04)	24,011 (31.14)	25,108 (31.14)
65-69	1,035 (30.85)	17,069 (22.09)	1,107 (31.32)	16,997 (22.04)	18,104 (22.45)
≥70	615 (18.33)	9,233 (11.95)	587 (16.61)	9,261 (12.01)	9,848 (12.21)
Missing	1 (0.03)	20 (0.03)	0 (0.0)	21 (0.03)	21 (0.03)
Sex - Female [n, (%)]	1,263 (37.63)	32,484 (42.02)	1,281 (36.25)	32,466 (42.1)	33,747 (41.84)
Missing	0 (0.0)	0 (0.0)	0 (0.0)	0 (0.0)	0 (0.0)
Ethnicity - White [n, (%)]	2,978 (88.74)	68,252 (88.34)	3,085 (87.32)	68,145 (88.4)	71,230 (88.36)
Missing	0 (0.0)	43 (0.06)	1 (0.03)	42 (0.05)	43 (0.05)
Highest qualification [n, (%)]					
Degree	777 (23.19)	25,126 (32.59)	838 (23.75)	25,065 (32.58)	25,903 (32.2)
Some college	776 (23.16)	17,978 (23.32)	815 (23.09)	17,939 (23.32)	18,754 (23.31)
Post-secondary school	1,341 (40.02)	27,394 (35.53)	1,393 (39.47)	27,342 (35.54)	28,735 (35.72)
Secondary school	414 (12.35)	5,726 (7.43)	423 (11.99)	5,717 (7.43)	6,140 (7.63)
None of the above	43 (1.28)	877 (1.14)	60 (1.7)	860 (1.12)	920 (1.14)
Missing	5 (0.15)	202 (0.26)	5 (0.14)	202 (0.26)	207 (0.26)
Body mass index					
<18.5	44 (1.33)	562 (0.74)	50 (1.43)	556 (0.73)	606 (0.76)
18.5-24	1,258 (38.03)	24,084 (31.65)	1,286 (36.9)	24,056 (31.68)	25,342 (31.91)
25-29	1,387 (41.93)	33,013 (43.38)	1,471 (42.21)	32,929 (43.37)	34,400 (43.32)
30-34	482 (14.57)	13,249 (17.41)	510 (14.63)	13,221 (17.41)	13,731 (17.29)
≥35	137 (4.14)	5,194 (6.83)	168 (4.82)	5,163 (6.8)	5,331 (6.71)
Missing	48 (1.43)	1201 (1.55)	49 (1.39)	1200 (1.56)	1249 (1.55)
Smoking status					
Former	1,840 (54.83)	62,769 (81.2)	1,910 (54.05)	62,699 (81.3)	64,609 (80.1)
Current	1,516 (45.17)	14,534 (18.8)	1,624 (45.95)	14,426 (18.7)	16,050 (19.9)
Missing	0 (0.0)	0 (0.0)	0 (0.0)	0 (0.0)	0 (0.0)
Age started smoking					
<16	865 (26.07)	14,583 (18.98)	880 (25.21)	14,568 (19.0)	15,448 (19.27)
16-20	1,936 (58.35)	46,507 (60.52)	2,058 (58.95)	46,385 (60.5)	48,443 (60.43)
>20	517 (15.58)	15,755 (20.5)	553 (15.84)	15,719 (20.5)	16,272 (20.3)
Missing	38 (1.13)	458 (0.59)	43 (1.22)	453 (0.59)	496 (0.61)
Years smoked					
<10	51 (1.55)	9,171 (12.12)	48 (1.38)	9,174 (12.15)	9,222 (11.67)
10-19	179 (5.43)	15,358 (20.29)	196 (5.65)	15,341 (20.31)	15,537 (19.67)
20-29	344 (10.44)	16,048 (21.2)	386 (11.13)	16,006 (21.19)	16,392 (20.75)
30-39	803 (24.37)	18,008 (23.79)	842 (24.28)	17,969 (23.79)	18,811 (23.81)
≥40	1,918 (58.21)	17,110 (22.6)	1,996 (57.55)	17,032 (22.55)	19,028 (24.09)
Missing	61 (1.82)	1608 (2.08)	66 (1.87)	1603 (2.08)	1669 (2.07)

Continued…

Cigarettes per day [n, (%)]					
1-10	379 (11.33)	20,249 (26.26)	404 (11.46)	20,224 (26.29)	20,628 (25.64)
11-20	1,155 (34.54)	28,180 (36.54)	1,247 (35.37)	28,088 (36.51)	29,335 (36.46)
21-30	894 (26.73)	15,131 (19.62)	953 (27.03)	15,072 (19.59)	16,025 (19.92)
31-40	555 (16.6)	8,285 (10.74)	558 (15.83)	8,282 (10.76)	8,840 (10.99)
>40	361 (10.8)	5,274 (6.84)	364 (10.32)	5,271 (6.85)	5,635 (7.0)
Missing	12 (0.36)	184 (0.24)	8 (0.23)	188 (0.24)	196 (0.24)
Pack-years of smoking [n, (%)]					
<10	88 (2.68)	12,874 (17.04)	86 (2.48)	12,876 (17.08)	12,962 (16.44)
10-19	196 (5.97)	14,648 (19.39)	219 (6.33)	14,625 (19.4)	14,844 (18.83)
20-29	320 (9.74)	11,382 (15.06)	356 (10.29)	11,346 (15.05)	11,702 (14.84)
30-39	328 (9.98)	9,850 (13.04)	341 (9.85)	9,837 (13.05)	10,178 (12.91)
≥40	2,353 (71.63)	26,805 (35.48)	2,459 (71.05)	26,699 (35.42)	29,158 (36.98)
Missing	71 (2.12)	1744 (2.26)	73 (2.07)	1742 (2.26)	1815 (2.25)
Personal history of cancer [n, (%)]	216 (6.44)	3,480 (4.5)	220 (6.23)	3,476 (4.51)	3,696 (4.58)
Missing	0 (0.0)	17 (0.02)	0 (0.0)	17 (0.02)	17 (0.02)
Chronic Obstructive Pulmonary Disease (COPD) / Emphysema / Bronchitis [n, (%)]	607 (18.09)	6,615 (8.56)	584 (16.53)	6,638 (8.61)	7,222 (8.95)
Missing	0 (0.0)	0 (0.0)	0 (0.0)	0 (0.0)	0 (0.0)
Family history of lung cancer [n, (%)]	562 (17.73)	8,479 (11.39)	573 (17.2)	8,468 (11.4)	9,041 (11.65)
Missing	187 (5.57)	2891 (3.74)	202 (5.72)	2876 (3.73)	3078 (3.82)

Table H: Outcomes by dataset

Years	UK Biobank		NLST control arm		PLCO ever-smokers (intervention arm)		PLCO ever-smokers (all)	
	Lung cancers (n)	Deaths from lung cancer (n)	Lung cancers (n)	Deaths from lung cancer (n)	Lung cancers (n)	Deaths from lung cancer (n)	Lung cancers (n)	Deaths from lung cancer (n)
1	202	55	185	36	169	32	261	55
2	439	194	314	105	281	105	489	195
3	712	356	442	186	412	188	747	358
4	1010	548	572	276	543	271	993	534
5	1335	737	719	365	643	351	1231	727
6	1653	956	885	481	754	445	1464	914
7	2060	1189	957	545	915	538	1744	1106
8	2456	1463	959	-	1046	655	2019	1329
9	2790	1722	-	-	1184	777	2303	1566
10	3112	1930	-	-	1330	881	2573	1770

Abbreviations: NLST, National Lung Screening Trial; PLCO, Prostate, Lung, Colorectal, and Ovarian Cancer Screening Trial.

UK Biobank and the US NLST control arm were model development datasets. External validation occurred amongst ever-smokers in the PLCO intervention arm and amongst all ever-smokers in the PLCO.

Details of UCL-D

UCL-D is an ensemble of four modelling pipelines predicting the five-year risk of dying from lung cancer. The final ensemble, constituent pipelines, and the weighting assigned to each pipeline are shown in Fig H. The tuned hyperparameters for the AdaBoost and LightGBM algorithms are shown in Table I.

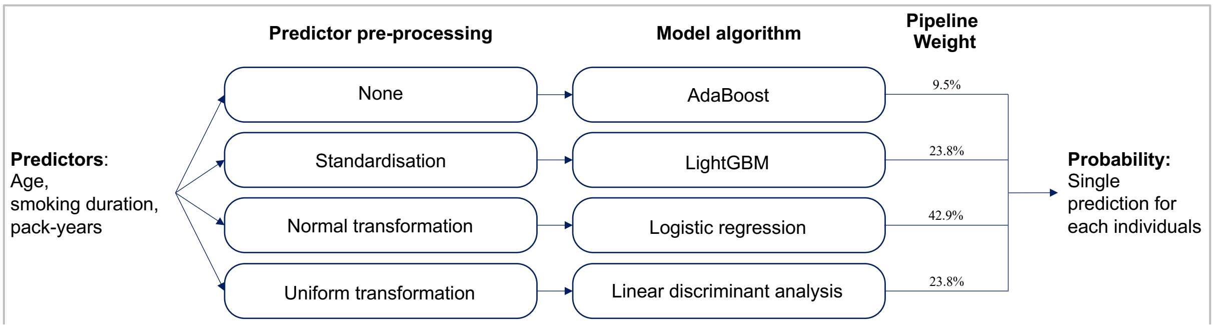

Fig H: The UCL-D ensemble and constituent pipelines (predicted outcome is five-year risk of death from lung cancer).

The three predictors are passed to each pipeline separately. The five-year probability of death from lung cancer that is outputted from each pipeline is then weighted, giving a single predicted risk for an individual. As an example, the first row shows the AdaBoost algorithm whilst the second pipeline includes a step where the variables are standardised before being passed to LightGBM.

Table I: Hyperparameters for the AdaBoost and LightGBM machine learning algorithms in UCL-D

Algorithm	Hyperparameter	Value
AdaBoost [6–8]	n_estimators	10
	learning_rate	0.1
LightGBM [11,12]	boosting_type	'gbdt'
	learning_rate	0.1
	max_depth	6
	reg_alpha	9.65x10-6
	reg_lambda	1.07x10-8
	colsample_by_tree	0.48
	subsample	0.61
	num_leaves	3
	min_child_samples	139

Details of UCL-I

UCL-I is also an ensemble of four modelling pipelines that instead predicts the five-year risk of lung cancer occurrence. Details of the ensemble and tuned hyperparameters for the constituent machine learning algorithms are shown in Fig I and Table J.

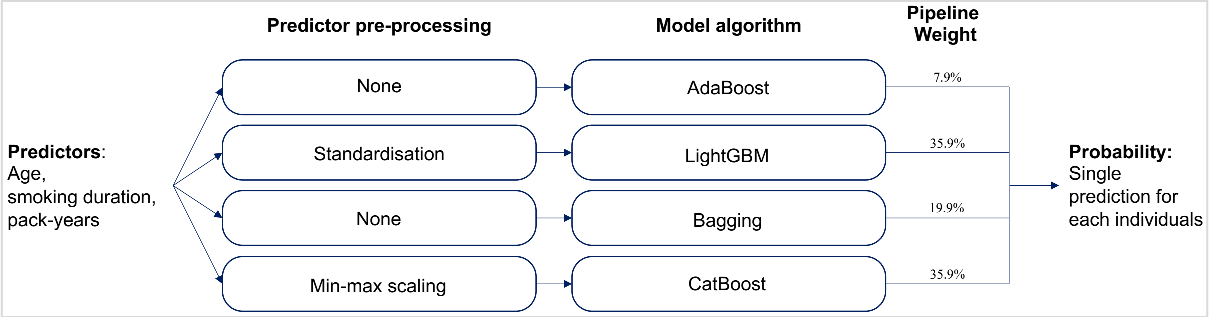

Fig I: Details of the UCL-I ensemble (predicted outcome is five-year risk of developing lung cancer).

Table J: Hyperparameters for the AdaBoost, LightGBM, and CatBoost machine learning algorithms in UCL-I

Algorithm	Hyperparameter	Value
AdaBoost [6–8]	n_estimators	10
	learning_rate	0.1
LightGBM [11,12]	boosting_type	'gbdt'
	learning_rate	0.1
	max_depth	6
	reg_alpha	0.036
	reg_lambda	9.08x10-5
	colsample_by_tree	0.11
	subsample	0.11
	num_leaves	3
	min_child_samples	23
CatBoost [9,10]	learning_rate	0.039
	depth	7
	l2_leaf_reg	554.36
	random_strength	1.303
	grow_policy	"Lossguide"

Table K: Discrimination (AUC) of models amongst ever-smokers in the UK Biobank

	AUC with 95% confidence intervals					
	UCL-D	LCDRAT	UCL-I	LCRAT	PLCOm2012	LLPv2
Overall	0.826 (0.815, 0.838)	0.829 (0.813, 0.841)	0.810 (0.800, 0.820)	0.815 (0.805, 0.827)	0.797 (0.783, 0.81)	0.779 (0.767, 0.793)
Age						
40-49	0.747 (0.659, 0.838)	0.755 (0.616, 0.904)	0.781 (0.727, 0.834)	0.793 (0.692, 0.865)	0.797 (0.721, 0.876)	0.672 (0.575, 0.775)
50-59	0.807 (0.780, 0.834)	0.803 (0.769, 0.834)	0.777 (0.754, 0.799)	0.781 (0.751, 0.808)	0.779 (0.751, 0.81)	0.719 (0.687, 0.748)
60-72	0.788 (0.772, 0.802)	0.792 (0.769, 0.805)	0.769 (0.756, 0.781)	0.776 (0.762, 0.791)	0.765 (0.750, 0.780)	0.740 (0.725, 0.754)
Sex						
Female	0.830 (0.812, 0.846)	0.825 (0.798, 0.844)	0.812 (0.798, 0.825)	0.811 (0.793, 0.831)	0.796 (0.780, 0.817)	0.771 (0.750, 0.791)
Male	0.820 (0.805, 0.838)	0.829 (0.808, 0.845)	0.809 (0.796, 0.821)	0.819 (0.802, 0.831)	0.798 (0.781, 0.815)	0.783 (0.767, 0.797)
Smoking status						
Former	0.815 (0.796, 0.833)	0.813 (0.792, 0.834)	0.794 (0.780, 0.808)	0.798 (0.783, 0.816)	0.778 (0.760, 0.798)	0.775 (0.757, 0.794)
Current	0.773 (0.751, 0.793)	0.780 (0.759, 0.802)	0.778 (0.763, 0.792)	0.787 (0.773, 0.801)	0.767 (0.751, 0.781)	0.743 (0.726, 0.757)
Ethnicity						
Other	0.818 (0.722, 0.982)	0.806 (0.631, 0.972)	0.810 (0.740, 0.889)	0.789 (0.660, 0.862)	0.827 (0.755, 0.905)	0.798 (0.737, 0.857)
White	0.825 (0.812, 0.837)	0.827 (0.813, 0.840)	0.809 (0.799, 0.819)	0.815 (0.805, 0.827)	0.796 (0.781, 0.809)	0.778 (0.765, 0.791)
Household income (GBP, £)						
<18,000	0.786 (0.764, 0.802)	0.791 (0.768, 0.811)	0.769 (0.755, 0.785)	0.782 (0.762, 0.800)	0.766 (0.747, 0.785)	0.742 (0.722, 0.759)
18,000-30,999	0.816 (0.791, 0.837)	0.812 (0.787, 0.836)	0.794 (0.777, 0.814)	0.803 (0.781, 0.822)	0.785 (0.762, 0.805)	0.749 (0.722, 0.771)
31,000-51,999	0.811 (0.780, 0.848)	0.822 (0.772, 0.861)	0.791 (0.764, 0.816)	0.788 (0.752, 0.824)	0.771 (0.733, 0.807)	0.757 (0.719, 0.799)
52,000-100,000	0.836 (0.789, 0.877)	0.828 (0.763, 0.883)	0.821 (0.785, 0.853)	0.808 (0.755, 0.852)	0.798 (0.741, 0.851)	0.790 (0.736, 0.835)
>100,000	0.744 (0.614, 0.938)	0.756 (0.536, 0.924)	0.808 (0.733, 0.876)	0.772 (0.634, 0.875)	0.738 (0.583, 0.849)	0.755 (0.624, 0.850)

Abbreviations: AUC, area under the receiver operating curve; GBP, British pounds; LLPv2, Liverpool Lung Project model version 2; UCL-D predicts lung cancer death; UCL-I predicts occurrence of lung cancer; LCDRAT, Lung Cancer Death Risk Assessment Tool; LCRAT, Lung Cancer Risk Assessment Tool.
UCL-D and LCDRAT predict lung cancer death; remaining comparator models predict lung cancer occurrence.

Table L: Brier scores in the PLCO chest radiography arm

	Brier scores with 95% confidence intervals					
	UCL-D	LCDRAT	UCL-I	LCRAT	PLCOm2012*	LLPv2
Overall	0.0084 (0.0075, 0.0093)	0.0084 (0.0075, 0.0093)	0.0153 (0.0142, 0.0164)	0.0152 (0.0143, 0.0164)	0.0153 (0.0143, 0.0164)	0.0153 (0.0143, 0.0165)
Age category						
55-59	0.0046 (0.0037, 0.0057)	0.0046 (0.0036, 0.0057)	0.0090 (0.0077, 0.0105)	0.0090 (0.0077, 0.0105)	0.0090 (0.0077, 0.0105)	0.0091 (0.0078, 0.0106)
60-64	0.0068 (0.0059, 0.0081)	0.0069 (0.0059, 0.0082)	0.0127 (0.0110, 0.0147)	0.0128 (0.0111, 0.0147)	0.0128 (0.0111, 0.0147)	0.0128 (0.0110, 0.0148)
65-69	0.0122 (0.0100, 0.0146)	0.0122 (0.0100, 0.0145)	0.0224 (0.0198, 0.0257)	0.0223 (0.0197, 0.0254)	0.0222 (0.0197, 0.0253)	0.0225 (0.0199, 0.0259)
70-74	0.0160 (0.0129, 0.0193)	0.0161 (0.0129, 0.0193)	0.0261 (0.0219, 0.0298)	0.0263 (0.0221, 0.0299)	0.0265 (0.0226, 0.0301)	0.0263 (0.0220, 0.0300)
Sex						
Female	0.0071 (0.0058, 0.0083)	0.0072 (0.0059, 0.0084)	0.0138 (0.0125, 0.0155)	0.0138 (0.0125, 0.0155)	0.0139 (0.0125, 0.0155)	0.0139 (0.0125, 0.0156)
Male	0.0093 (0.0082, 0.0107)	0.0093 (0.0082, 0.0107)	0.0163 (0.0150, 0.0180)	0.0162 (0.0149, 0.0180)	0.0163 (0.0150, 0.0180)	0.0163 (0.0151, 0.0182)
Smoking status						
Former	0.0058 (0.0050, 0.0066)	0.0059 (0.0050, 0.0066)	0.0108 (0.0098, 0.0118)	0.0108 (0.0098, 0.0119)	0.0109 (0.0099, 0.0119)	0.0109 (0.0099, 0.0119)
Current	0.0187 (0.0160, 0.0213)	0.0187 (0.0160, 0.0214)	0.0329 (0.0300, 0.0368)	0.0329 (0.0299, 0.0366)	0.0330 (0.0300, 0.0368)	0.0331 (0.0301, 0.0371)
Qualifications						
Degree	0.0060 (0.0047, 0.0072)	0.0060 (0.0047, 0.0071)	0.0109 (0.0092, 0.0126)	0.0109 (0.0091, 0.0126)	0.0109 (0.0091, 0.0125)	0.0110 (0.0092, 0.0127)
Some college	0.0076 (0.0061, 0.0095)	0.0076 (0.0061, 0.0095)	0.0144 (0.0122, 0.0165)	0.0144 (0.0122, 0.0165)	0.0144 (0.0122, 0.0165)	0.0145 (0.0123, 0.0166)
Post-secondary	0.0091 (0.0077, 0.0105)	0.0091 (0.0077, 0.0105)	0.0166 (0.0148, 0.0187)	0.0165 (0.0147, 0.0187)	0.0166 (0.0148, 0.0187)	0.0167 (0.0148, 0.0189)
Secondary school	0.0157 (0.0119, 0.0200)	0.0158 (0.0121, 0.0201)	0.0273 (0.0222, 0.0324)	0.0275 (0.0225, 0.0325)	0.0276 (0.0227, 0.0324)	0.0275 (0.0222, 0.0326)
None of above	0.0210 (0.0102, 0.0391)	0.0210 (0.0103, 0.0387)	0.0315 (0.0146, 0.0492)	0.0320 (0.0153, 0.0496)	0.0322 (0.0158, 0.0494)	0.0314 (0.0144, 0.0490)
Ethnicity						
Asian	0.0069 (0.0031, 0.0109)	0.0070 (0.0030, 0.0110)	0.0111 (0.0056, 0.0172)	0.0111 (0.0056, 0.0174)	0.0112 (0.0057, 0.0174)	0.0112 (0.0057, 0.0173)
Black	0.0159 (0.0109, 0.0209)	0.0158 (0.0109, 0.0205)	0.0258 (0.0196, 0.0316)	0.0256 (0.0197, 0.0313)	0.0255 (0.0197, 0.0309)	0.0257 (0.0196, 0.0316)
Other	0.0106 (0.0058, 0.0166)	0.0105 (0.0057, 0.0165)	0.0146 (0.0091, 0.0206)	0.0146 (0.0090, 0.0205)	0.0150 (0.0095, 0.0209)	0.0145 (0.0089, 0.0202)
White	0.0079 (0.0070, 0.0088)	0.0079 (0.0070, 0.0089)	0.0148 (0.0136, 0.0159)	0.0147 (0.0135, 0.0158)	0.0148 (0.0136, 0.0158)	0.0149 (0.0137, 0.0160)

Abbreviations: PLCO, Prostate, Lung, Colorectal, and Ovarian (PLCO) Cancer Trial. LLP v2, Liverpool Lung Project model version 2.
UCL-D predicts lung cancer death; UCL-I predicts occurrence of lung cancer; LCDRAT, Lung Cancer Death Risk Assessment Tool;
LCRAT, Lung Cancer Risk Assessment Tool.
* The PLCOm2012 score predicts 6-year risk of developing lung cancer, whilst all other scores predict risk at 5-years. Brier scores vary by prevalence, so results presented here are for PLCOm2012 against 5-year outcomes to allow for direct comparison. Lower Brier scores indicate better model performance.
UCL-D and LCDRAT predict lung cancer death; remaining comparator models predict lung cancer occurrence.

Table M: Brier scores of models amongst ever-smokers in the UK Biobank

	Brier scores with 95% confidence intervals					
	UCL-D	LCDRAT	UCL-I	LCRAT	PLCOm2012*	LLPv2
Overall	0.0034 (0.0031, 0.0036)	0.0034 (0.0031, 0.0036)	0.006 (0.0058, 0.0064)	0.006 (0.0057, 0.0063)	0.006 (0.0057, 0.0063)	0.0062 (0.0059, 0.0065)
Age category						
40-49	0.0005 (0.0002, 0.0007)	0.0005 (0.0002, 0.0007)	0.0009 (0.0006, 0.0012)	0.0009 (0.0006, 0.0013)	0.0009 (0.0006, 0.0013)	0.0009 (0.0006, 0.0013)
50-59	0.0025 (0.0022, 0.0029)	0.0025 (0.0021, 0.0028)	0.0043 (0.0038, 0.0047)	0.0042 (0.0038, 0.0048)	0.0042 (0.0038, 0.0048)	0.0042 (0.0038, 0.0049)
60-72	0.0052 (0.0047, 0.0056)	0.0051 (0.0048, 0.0056)	0.0094 (0.0088, 0.0100)	0.0093 (0.0090, 0.0099)	0.0094 (0.0090, 0.0099)	0.0097 (0.0093, 0.0102)
Sex						
Female	0.0029 (0.0026, 0.0031)	0.0028 (0.0025, 0.0032)	0.0056 (0.0051, 0.0060)	0.0055 (0.0050, 0.0059)	0.0055 (0.0050, 0.0059)	0.0056 (0.0050, 0.0059)
Male	0.0039 (0.0035, 0.0042)	0.0038 (0.0035, 0.0042)	0.0065 (0.0061, 0.0070)	0.0065 (0.0061, 0.0069)	0.0065 (0.0061, 0.0069)	0.0068 (0.0063, 0.0072)
Smoking status						
Former	0.0023 (0.0020, 0.0025)	0.0022 (0.0020, 0.0025)	0.0043 (0.0041, 0.0046)	0.0043 (0.0040, 0.0046)	0.0043 (0.0040, 0.0046)	0.0044 (0.0042, 0.0047)
Current	0.0069 (0.0061, 0.0076)	0.0069 (0.0062, 0.0075)	0.0115 (0.0107, 0.0124)	0.0115 (0.0107, 0.0123)	0.0115 (0.0107, 0.0123)	0.0117 (0.0109, 0.0125)
Ethnicity						
Other	0.0017 (0.0006, 0.0023)	0.0015 (0.0008, 0.0023)	0.0031 (0.0019, 0.0042)	0.0029 (0.0020, 0.0041)	0.0029 (0.0020, 0.0040)	0.0030 (0.0022, 0.0042)
White	0.0034 (0.0032, 0.0037)	0.0034 (0.0032, 0.0037)	0.0062 (0.0059, 0.0065)	0.0061 (0.0058, 0.0064)	0.0062 (0.0059, 0.0065)	0.0063 (0.0060, 0.0066)
Household income (GBP £)						
<18,000	0.0059 (0.0052, 0.0064)	0.0059 (0.0052, 0.0065)	0.0104 (0.0096, 0.0112)	0.0103 (0.0095, 0.0112)	0.0103 (0.0095, 0.0113)	0.0105 (0.0097, 0.0115)
18,000 to 30,999	0.0038 (0.0033, 0.0042)	0.0037 (0.0033, 0.0042)	0.0068 (0.0062, 0.0074)	0.0068 (0.0061, 0.0075)	0.0068 (0.0062, 0.0075)	0.0070 (0.0064, 0.0077)
31,000 to 51,999	0.0019 (0.0015, 0.0021)	0.0018 (0.0015, 0.0022)	0.0034 (0.0029, 0.0039)	0.0033 (0.0029, 0.0039)	0.0033 (0.0029, 0.0039)	0.0035 (0.0030, 0.0040)
52,000 to 100,000	0.0013 (0.0009, 0.0016)	0.0013 (0.0010, 0.0017)	0.0024 (0.0019, 0.0029)	0.0024 (0.0019, 0.0029)	0.0024 (0.0019, 0.0029)	0.0024 (0.0020, 0.0030)
>100,000	0.0012 (0.0003, 0.0017)	0.0011 (0.0006, 0.0018)	0.0021 (0.0011, 0.0029)	0.0020 (0.0013, 0.0031)	0.0020 (0.0013, 0.0031)	0.0021 (0.0013, 0.0032)

Abbreviations: GBP, British pounds; LLPv2, Liverpool Lung Project model version 2; UCL-D predicts lung cancer death; UCL-I predicts occurrence of lung cancer; LCDRAT, Lung Cancer Death Risk Assessment Tool; LCRAT, Lung Cancer Risk Assessment Tool.
* The PLCOm2012 score predicts 6-year risk of developing lung cancer, whilst all other scores predict risk at 5-years. Brier scores vary by prevalence, so results presented here are for PLCOm2012 against 5-year outcomes to allow for direct comparison. Lower Brier scores indicate better model performance. UCL-D and LCDRAT predict lung cancer death; remaining comparator models predict lung cancer occurrence.

Table N: Calibration of models amongst ever-smokers in the UK Biobank

	Ratio of expected-to-observed cancers with 95% confidence intervals					
	UCL-D	LCDRAT	UCL-I	LCRAT	PLCOm2012	LLPv2
Overall	1.47 (1.38, 1.58)	1.44 (1.34, 1.54)	1.35 (1.28, 1.42)	1.36 (1.30, 1.44)	1.06 (1.02, 1.11)	1.97 (1.89, 2.08)
Age						
40-49	3.32 (0.23, 4.55)	1.88 (1.28, 4.17)	1.98 (1.30, 2.55)	2.38 (1.69, 3.46)	1.71 (1.31, 2.27)	0.92 (0.66, 1.35)
50-59	1.43 (1.18, 1.59)	1.22 (1.10, 1.44)	1.38 (1.21, 1.53)	1.40 (1.22, 1.55)	1.06 (0.94, 1.17)	1.41 (1.23, 1.57)
60-72	1.42 (1.32, 1.54)	1.49 (1.37, 1.62)	1.31 (1.23, 1.39)	1.31 (1.24, 1.38)	1.03 (0.98, 1.08)	2.20 (2.07, 2.31)
Sex						
Female	1.48 (1.37, 1.63)	1.37 (1.23, 1.57)	1.24 (1.14, 1.33)	1.27 (1.18, 1.40)	0.97 (0.91, 1.07)	1.54 (1.44, 1.71)
Male	1.46 (1.32, 1.59)	1.49 (1.36, 1.61)	1.44 (1.32, 1.53)	1.43 (1.34, 1.54)	1.12 (1.06, 1.20)	2.31 (2.17, 2.49)
Smoking status						
Previous	1.72 (1.57, 1.90)	1.50 (1.33, 1.63)	1.51 (1.40, 1.61)	1.31 (1.22, 1.40)	1.11 (1.05, 1.19)	2.39 (2.24, 2.56)
Current	1.22 (1.09, 1.35)	1.38 (1.27, 1.55)	1.16 (1.06, 1.25)	1.42 (1.33, 1.53)	1.00 (0.93, 1.08)	1.48 (1.39, 1.61)
Highest qualification						
Degree	2.23 (1.63, 2.65)	1.74 (1.45, 2.19)	1.95 (1.65, 2.20)	1.71 (1.51, 1.96)	1.22 (1.09, 1.35)	2.97 (2.62, 3.42)
Some college	1.44 (0.97, 1.70)	1.19 (0.95, 1.49)	1.38 (1.09, 1.60)	1.23 (1.06, 1.46)	1.01 (0.85, 1.19)	2.17 (1.88, 2.56)
Post-secondary	1.59 (1.17, 1.83)	1.52 (1.32, 1.89)	1.44 (1.21, 1.61)	1.44 (1.27, 1.66)	1.13 (0.99, 1.29)	2.11 (1.86, 2.43)
Secondary school	1.74 (1.48, 1.99)	1.73 (1.46, 2.04)	1.50 (1.32, 1.67)	1.60 (1.42, 1.81)	1.17 (1.08, 1.31)	2.12 (1.89, 2.40)
None of the above	1.11 (1.00, 1.21)	1.26 (1.14, 1.42)	1.06 (0.97, 1.13)	1.16 (1.07, 1.26)	0.95 (0.87, 1.02)	1.54 (1.42, 1.68)
Ethnicity						
Other	2.01 (0.01, 2.99)	1.80 (1.14, 3.38)	1.67 (0.73, 2.19)	1.60 (1.10, 2.27)	1.15 (0.86, 1.54)	2.62 (1.80, 3.85)
White	1.46 (1.36, 1.57)	1.43 (1.34, 1.53)	1.34 (1.27, 1.41)	1.36 (1.29, 1.43)	1.06 (1.01, 1.11)	1.97 (1.87, 2.07)
Household income (GBP, £)						
<18,000	1.25 (1.13, 1.39)	1.32 (1.19, 1.49)	1.17 (1.07, 1.26)	1.23 (1.12, 1.34)	0.99 (0.91, 1.06)	1.69 (1.54, 1.84)
18,000-30,999	1.38 (1.20, 1.54)	1.35 (1.21, 1.55)	1.26 (1.15, 1.37)	1.26 (1.14, 1.39)	0.98 (0.90, 1.06)	1.94 (1.76, 2.14)
31,000-51,999	2.04 (1.68, 2.39)	1.86 (1.50, 2.31)	1.84 (1.52, 2.08)	1.80 (1.54, 2.08)	1.34 (1.17, 1.52)	2.72 (2.30, 3.15)
52,000-100,000	2.23 (1.51, 2.72)	1.84 (1.40, 2.40)	1.95 (1.44, 2.28)	1.87 (1.53, 2.34)	1.36 (1.11, 1.64)	2.72 (2.22, 3.39)
≥100,000	2.24 (1.38, 3.35)	1.98 (1.16, 3.88)	2.00 (0.50, 2.67)	1.91 (1.23, 3.20)	1.43 (0.94, 2.30)	2.86 (1.86, 4.80)

Abbreviations: GBP, British pounds; LLPv2, Liverpool Lung Project model version 2; UCL-D predicts lung cancer death; UCL-I predicts occurrence of lung cancer; LCDRAT, Lung Cancer Death Risk Assessment Tool; LCRAT, Lung Cancer Risk Assessment Tool.

Table O: Model sensitivity and sensitivity at specified risk thresholds in the PLCO dataset

	Risk threshold (%)	Sensitivity (95% CI)	Specificity (95% CI)
Predicting 5-year risk of death from lung cancer			
UCL-D	0.68	0.855 (0.828, 0.882)	0.574 (0.570, 0.577)
USPSTF-2021	-	0.775 (0.746, 0.809)	0.574 (0.570, 0.578)
Predicting 5-year risk of developing lung cancer			
UCL-I	1.17	0.839 (0.820, 0.861)	0.577 (0.574, 0.580)
USPSTF-2021	-	0.777 (0.758, 0.802)	0.576 (0.572, 0.579)

Abbreviations: 95% CI, 95% confidence intervals; PLCO, Prostate, Lung, Colorectal, and Ovarian (PLCO) Cancer Trial; USPSTF-2021, US Preventive Services Taskforce 2021 screening eligibility criteria.
Risk thresholds set using a fixed population approach at a level that would screen an equivalent number as the USPSTF-2021 in the PLCO external validation dataset. The entire PLCO dataset was used for these analyses.

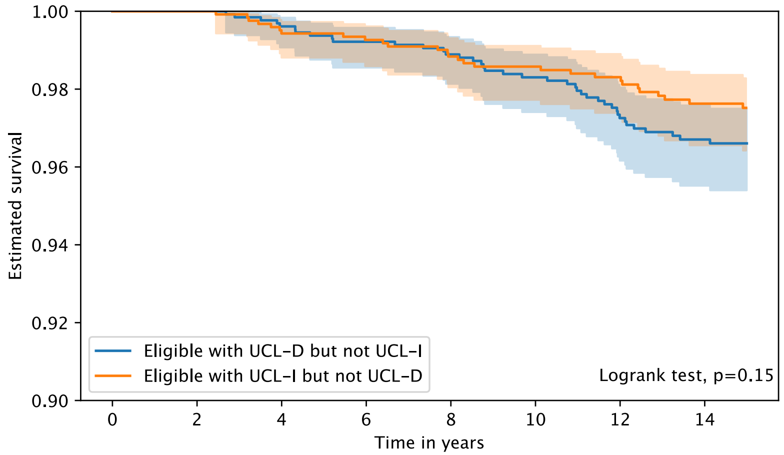

**Eligible with UCL-D but not UCL-I**								
At risk	1313	1299	1274	1239	1198	1150	1095	973
Censored	1	15	35	65	102	143	186	302
Events	0	0	5	10	14	21	33	39
**Eligible with UCL-I but not UCL-D**								
At risk	1253	1245	1213	1186	1151	1110	1041	941
Censored	0	8	33	58	88	126	192	285
Events	0	0	7	9	14	17	20	27

(a)

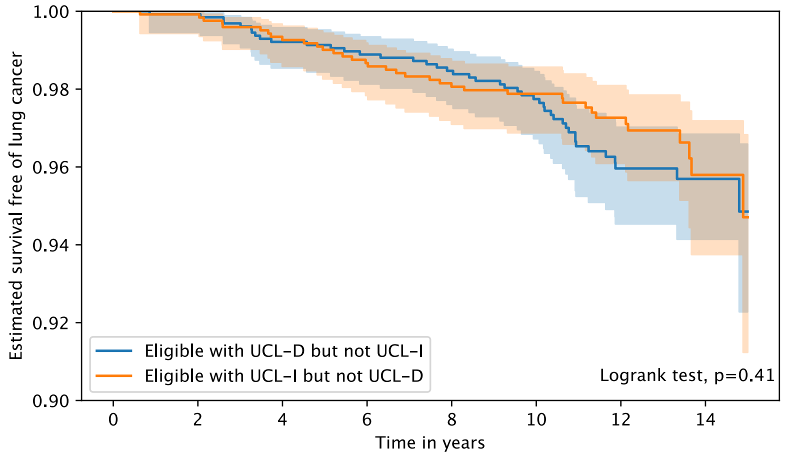

**Eligible with UCL-D but not UCL-I**								
At risk	1306	1284	1245	1204	1156	993	618	219
Censored	8	29	59	96	139	294	654	1052
Events	0	1	10	14	19	27	42	43
**Eligible with UCL-I but not UCL-D**								
At risk	1246	1230	1191	1156	1113	971	622	182
Censored	7	22	53	81	117	257	601	1036
Events	0	1	9	16	23	25	30	35

(b)

Fig J: Outcomes by eligibility for either UCL-D or UCL-I, but not both UCL models

Kaplan-Meier plots showing lung cancer deaths (a) and lung cancers (b) amongst the 1,314 participants in the Prostate, Lung, Colorectal, and Ovarian Cancer Screening (PLCO) Trial who would be eligible for screening with UCL-D (assuming a cut-off of 0.68%) but not UCL-I (assuming a cut-off of 1.17%) [blue lines] and amongst the 1,253 individuals who would be eligible for screening with UCL-I but not UCL-D [orange lines]. Shaded areas refer to 95% confidence intervals for each respective Kaplan-Meier curve. Overall, 34,756 individuals in the PLCO trial would have been eligible for screening with UCL-D at a cut-off of 0.68% and 34,695 with a cut-off of 1.17%. There is a trend shown in (a) towards those who would have been eligible for UCL-D but not UCL-I having a lower survival beyond 10 years (i.e., more deaths from lung cancer), but the difference did not reach statistical significance.
Abbreviations: UCL-D predicts lung cancer death; UCL-I predicts occurrence of lung cancer.

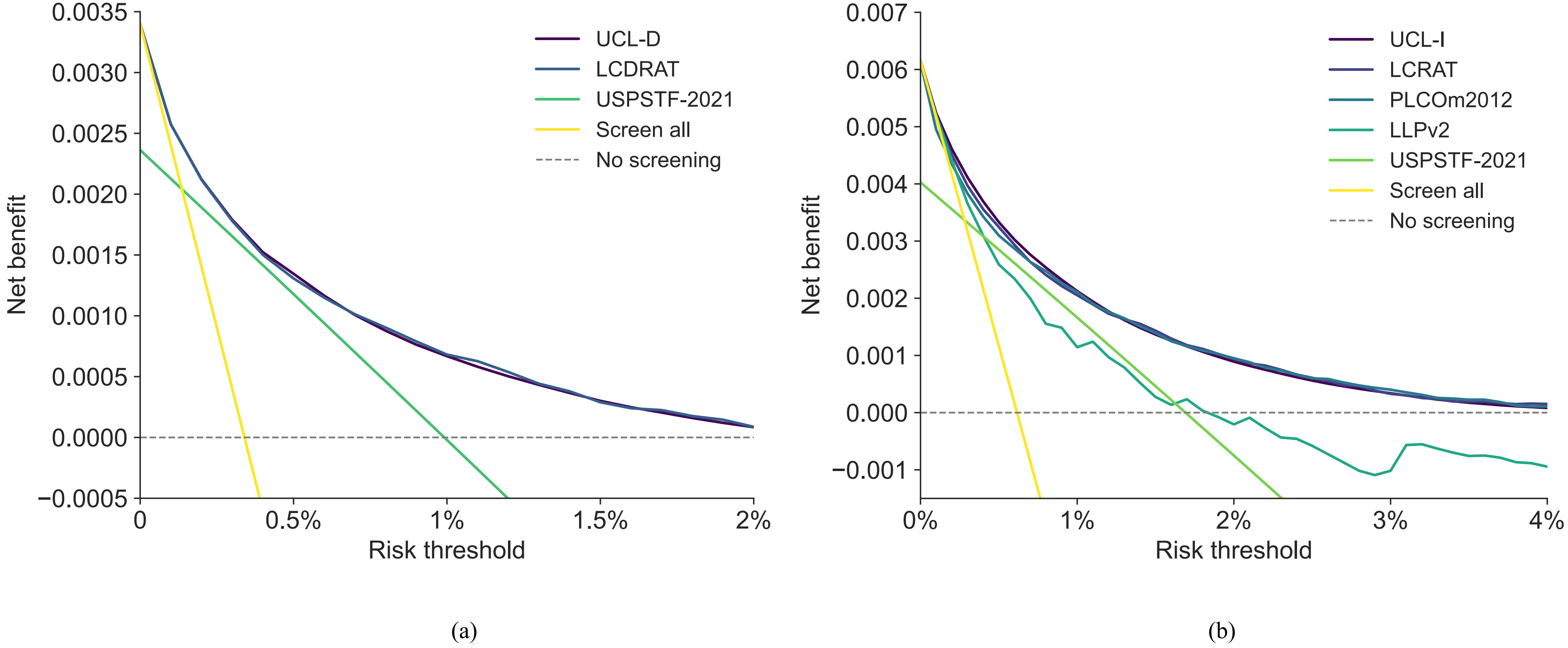

Fig K: Net benefit of models in the UK Biobank

Net benefit across a range of thresholds of models predicting 5-year risk of death from lung cancer (A) and developing lung cancer (B) compared against US Preventive Services Taskforce (USPSTF)-2021 screening eligibility criteria in the UK Biobank dataset. Risk model-based approaches had a higher net benefit than screening either all men or using the USPSTF-2021 criteria to determine screening eligibility. The PLCOm2012 was originally fitted to predict 6-year risk of lung cancer; to make comparison possible, here net benefit was calculated based on predicting 5-year risk of lung cancer. The performance of PLCOm2012 over a 5-year timeframe was equivalent to that of a 6-year timeframe in the UK Biobank with an AUC of 0.796 (0.782, 0.811) and a Brier score of 0.006 (0.0057, 0.0063). In this analysis, the difference in net benefit

between two approaches to selecting for screening at a particular risk threshold would be the change in true positives at that threshold level who would be screened given a fixed number of false positives [25].
Abbreviations: LLPv2, Liverpool Lung Project model version 2; UCL-D predicts lung cancer death; UCL-I predicts occurrence of lung cancer; LCDRAT, Lung Cancer Death Risk Assessment Tool; LCRAT, Lung Cancer Risk Assessment Tool; USPSTF, US Preventive Services Taskforce.

Full Models

We present analyses in the full (control and radiography arm together) of the PLCO dataset. Here we also include the 'full' models developed alongside UCL-D and UCL. Both models are machine learning ensembles that were developed on the combined UK Biobank-NLST dataset.

UCLFull-D

UCLFull-D is an eight-variable model that predicts five-year risk of death from lung cancer with the following predictors:

1. Age,
2. Smoking duration (years),
3. Pack-years,
4. Smoking intensity (number of cigarettes per day),
5. Quit-years,
6. Body-mass index,
7. Chronic obstructive pulmonary disease (COPD), and
8. Family history of lung cancer.

UCLFull – D = $(0.143 * AdaBoost) + (0.714 * Logistic\ Regression) + (0.143 * LightGBM)$

UCLFull-I

UCLFull-I is a six-variable model that predicts five-year risk of developing lung cancer using the following predictors:

1. Age,
2. Smoking duration (years),
3. Pack-years,
4. Body-mass index,
5. COPD, and
6. family history of lung cancer.

UCLFull-I=

$$(0.391 * CatBoost) + (0.391 * Logistic\ Regression) + (0.087 * AdaBoost) + (0.130 * LightGBM)$$

Table P: Discriminative accuracy (AUC) in the whole PLCO cohort

	AUC with 95% confidence intervals								
	UCLFull-D	UCL-D	LCDRAT	UCLFull-I	UCL-I	LCRAT	PLCOm2012	LLP v2	LLP v3
PLCO overall	0.80 (0.78, 0.81)	0.79 (0.78, 0.81)	0.80 (0.79, 0.82)	0.79 (0.78, 0.8)	0.78 (0.77, 0.79)	0.79 (0.78, 0.8)	0.79 (0.78, 0.8)	0.74 (0.73, 0.76)	0.74 (0.73, 0.76)
Age category									
55-59	0.82 (0.78, 0.84)	0.8 (0.77, 0.83)	0.82 (0.78, 0.85)	0.81 (0.78, 0.83)	0.79 (0.77, 0.82)	0.81 (0.79, 0.84)	0.8 (0.78, 0.82)	0.74 (0.71, 0.76)	0.74 (0.71, 0.77)
60-64	0.79 (0.76, 0.81)	0.79 (0.75, 0.81)	0.79 (0.76, 0.81)	0.78 (0.76, 0.80)	0.77 (0.75, 0.80)	0.78 (0.76, 0.80)	0.78 (0.77, 0.80)	0.74 (0.71, 0.76)	0.72 (0.70, 0.75)
65-69	0.77 (0.74, 0.80)	0.76 (0.73, 0.79)	0.78 (0.75, 0.80)	0.77 (0.74, 0.79)	0.76 (0.74, 0.78)	0.78 (0.75, 0.80)	0.78 (0.76, 0.80)	0.72 (0.70, 0.75)	0.73 (0.70, 0.75)
70-74	0.73 (0.70, 0.77)	0.73 (0.70, 0.77)	0.74 (0.71, 0.78)	0.73 (0.70, 0.76)	0.73 (0.70, 0.76)	0.73 (0.71, 0.76)	0.74 (0.71, 0.77)	0.69 (0.66, 0.72)	0.68 (0.65, 0.71)
Sex									
Female	0.81 (0.79, 0.83)	0.80 (0.78, 0.82)	0.81 (0.79, 0.84)	0.80 (0.78, 0.81)	0.78 (0.76, 0.8)	0.80 (0.78, 0.81)	0.80 (0.78, 0.81)	0.74 (0.72, 0.76)	0.74 (0.72, 0.76)
Male	0.79 (0.77, 0.81)	0.78 (0.76, 0.8)	0.80 (0.78, 0.82)	0.79 (0.77, 0.80)	0.78 (0.77, 0.8)	0.79 (0.78, 0.81)	0.79 (0.78, 0.81)	0.75 (0.73, 0.76)	0.75 (0.73, 0.76)
Smoking status									
Former	0.81 (0.78, 0.83)	0.80 (0.78, 0.82)	0.81 (0.79, 0.83)	0.79 (0.77, 0.81)	0.79 (0.77, 0.80)	0.79 (0.78, 0.81)	0.79 (0.78, 0.81)	0.75 (0.73, 0.77)	0.74 (0.73, 0.76)
Current	0.69 (0.66, 0.72)	0.67 (0.64, 0.70)	0.70 (0.67, 0.73)	0.69 (0.67, 0.71)	0.67 (0.65, 0.69)	0.70 (0.68, 0.72)	0.69 (0.67, 0.71)	0.64 (0.61, 0.66)	0.65 (0.62, 0.67)
Qualifications									
Degree	0.83 (0.80, 0.86)	0.83 (0.80, 0.86)	0.84 (0.81, 0.87)	0.82 (0.79, 0.84)	0.81 (0.78, 0.83)	0.82 (0.8, 0.84)	0.82 (0.8, 0.84)	0.77 (0.74, 0.79)	0.77 (0.74, 0.79)
Some college	0.79 (0.76, 0.82)	0.79 (0.75, 0.82)	0.78 (0.75, 0.81)	0.79 (0.77, 0.82)	0.79 (0.77, 0.81)	0.79 (0.76, 0.81)	0.80 (0.77, 0.82)	0.75 (0.73, 0.78)	0.74 (0.72, 0.77)
Post-secondary	0.79 (0.77, 0.82)	0.78 (0.75, 0.80)	0.79 (0.77, 0.81)	0.78 (0.76, 0.80)	0.77 (0.75, 0.79)	0.78 (0.76, 0.8)	0.77 (0.76, 0.79)	0.73 (0.71, 0.75)	0.73 (0.71, 0.75)
Secondary school	0.72 (0.67, 0.78)	0.71 (0.66, 0.77)	0.74 (0.68, 0.78)	0.72 (0.67, 0.76)	0.72 (0.67, 0.76)	0.74 (0.69, 0.77)	0.74 (0.70, 0.77)	0.68 (0.63, 0.72)	0.68 (0.63, 0.72)
None of above	0.68 (0.57, 0.78)	0.66 (0.53, 0.76)	0.70 (0.58, 0.81)	0.67 (0.56, 0.75)	0.66 (0.57, 0.75)	0.67 (0.55, 0.76)	0.68 (0.58, 0.77)	0.61 (0.51, 0.73)	0.62 (0.51, 0.74)
Ethnicity									
Asian	0.85 (0.79, 0.91)	0.86 (0.80, 0.91)	0.83 (0.76, 0.90)	0.74 (0.64, 0.84)	0.74 (0.62, 0.84)	0.74 (0.66, 0.83)	0.75 (0.67, 0.82)	0.71 (0.61, 0.81)	0.70 (0.59, 0.81)
Black	0.79 (0.73, 0.85)	0.78 (0.72, 0.84)	0.81 (0.75, 0.86)	0.78 (0.72, 0.83)	0.77 (0.72, 0.82)	0.79 (0.74, 0.83)	0.79 (0.75, 0.83)	0.75 (0.71, 0.80)	0.74 (0.70, 0.79)
Other	0.81 (0.72, 0.90)	0.78 (0.67, 0.87)	0.83 (0.76, 0.91)	0.80 (0.74, 0.86)	0.78 (0.71, 0.85)	0.81 (0.74, 0.87)	0.78 (0.70, 0.84)	0.73 (0.64, 0.83)	0.73 (0.64, 0.83)
White	0.80 (0.78, 0.81)	0.79 (0.78, 0.80)	0.80 (0.78, 0.81)	0.79 (0.78, 0.80)	0.78 (0.77, 0.79)	0.79 (0.78, 0.81)	0.80 (0.79, 0.81)	0.74 (0.73, 0.76)	0.74 (0.73, 0.76)

Abbreviations: AUC, area under the receiver operating curve; PLCO, Prostate, Lung, Colorectal, and Ovarian (PLCO) Cancer Trial; LLP v2 and v3 refer to the Liverpool Lung Project models versions 2 and 3; UCLFull-D and UCL-D predict risk of lung cancer death; UCLFull-I and UCL-I predict risk of lung cancer occurrence; LCDRAT, Lung Cancer Death Risk Assessment Tool; LCRAT, Lung Cancer Risk Assessment Tool.

Note that the LCDRAT, LCRAT, and PLCOm2012 models were developed in the control arm of the PLCO cohort. The relative performance of the UCL models is therefore notable.

Table Q: Overall performance (Brier scores) in the whole PLCO cohort

	Brier score with 95% confidence interval								
	UCLFull-D	UCL-D	LCDRAT	UCLFull-I	UCL-I	LCRAT	PLCOm2012*	LLP v2	LLP v3
PLCO overall	0.0088 (0.0083, 0.0094)	0.0089 (0.0083, 0.0094)	0.0088 (0.0083, 0.0094)	0.0147 (0.014, 0.0155)	0.0147 (0.0141, 0.0155)	0.0147 (0.0141, 0.0155)	0.0147 (0.0141, 0.0155)	0.0148 (0.0142, 0.0156)	0.0149 (0.0143, 0.0157)
Age category									
55-59	0.0048 (0.0042, 0.0056)	0.0048 (0.0042, 0.0056)	0.0048 (0.0041, 0.0055)	0.0087 (0.0077, 0.0099)	0.0087 (0.0077, 0.0099)	0.0086 (0.0077, 0.0098)	0.0087 (0.0077, 0.0098)	0.0088 (0.0077, 0.01)	0.0088 (0.0078, 0.01)
60-64	0.0074 (0.0063, 0.0082)	0.0074 (0.0063, 0.0082)	0.0074 (0.0063, 0.0082)	0.0128 (0.0114, 0.0139)	0.0128 (0.0115, 0.014)	0.0128 (0.0115, 0.014)	0.0128 (0.0115, 0.014)	0.0128 (0.0115, 0.014)	0.0129 (0.0115, 0.0141)
65-69	0.0127 (0.0113, 0.0143)	0.0127 (0.0113, 0.0144)	0.0127 (0.0113, 0.0143)	0.0207 (0.019, 0.0225)	0.0208 (0.0191, 0.0225)	0.0207 (0.019, 0.0224)	0.0207 (0.019, 0.0224)	0.021 (0.0192, 0.0228)	0.0212 (0.0193, 0.023)
70-74	0.0166 (0.0146, 0.0192)	0.0167 (0.0146, 0.0192)	0.0166 (0.0146, 0.0192)	0.0254 (0.0225, 0.0279)	0.0255 (0.0225, 0.028)	0.0255 (0.0226, 0.0281)	0.0258 (0.0228, 0.0283)	0.0256 (0.0227, 0.0281)	0.0258 (0.0227, 0.0284)
Sex									
Female	0.0072 (0.0065, 0.0081)	0.0072 (0.0065, 0.0081)	0.0073 (0.0065, 0.0081)	0.013 (0.0119, 0.0141)	0.013 (0.012, 0.0141)	0.013 (0.0119, 0.0141)	0.0131 (0.012, 0.0142)	0.0131 (0.0121, 0.0142)	0.0131 (0.0121, 0.0143)
Male	0.0099 (0.009, 0.0109)	0.0099 (0.009, 0.0109)	0.0099 (0.009, 0.0109)	0.0159 (0.0146, 0.017)	0.0159 (0.0146, 0.017)	0.0159 (0.0146, 0.017)	0.0159 (0.0146, 0.017)	0.016 (0.0147, 0.0171)	0.0161 (0.0148, 0.0173)
Smoking status									
Former	0.0062 (0.0057, 0.0068)	0.0062 (0.0057, 0.0068)	0.0062 (0.0057, 0.0068)	0.0104 (0.0096, 0.0111)	0.0104 (0.0096, 0.0111)	0.0104 (0.0096, 0.0111)	0.0104 (0.0096, 0.0111)	0.0104 (0.0096, 0.0111)	0.0104 (0.0097, 0.0111)
Current	0.0193 (0.0172, 0.021)	0.0194 (0.0173, 0.0211)	0.0194 (0.0173, 0.021)	0.0322 (0.0298, 0.0344)	0.0324 (0.03, 0.0346)	0.0323 (0.0298, 0.0344)	0.0324 (0.03, 0.0346)	0.0326 (0.0301, 0.0349)	0.033 (0.0304, 0.0354)
Qualifications									
Degree	0.0059 (0.0051, 0.0068)	0.0059 (0.0051, 0.0069)	0.0059 (0.0051, 0.0069)	0.0104 (0.0094, 0.0116)	0.0104 (0.0094, 0.0117)	0.0104 (0.0094, 0.0116)	0.0104 (0.0094, 0.0116)	0.0105 (0.0095, 0.0117)	0.0105 (0.0095, 0.0118)
Some college	0.0083 (0.0072, 0.0094)	0.0083 (0.0072, 0.0094)	0.0083 (0.0072, 0.0094)	0.0143 (0.0126, 0.0161)	0.0144 (0.0126, 0.0161)	0.0144 (0.0127, 0.0161)	0.0144 (0.0127, 0.0161)	0.0145 (0.0127, 0.0162)	0.0146 (0.0128, 0.0164)
Post-secondary	0.0099 (0.0088, 0.0112)	0.01 (0.0089, 0.0113)	0.0099 (0.0088, 0.0112)	0.0162 (0.015, 0.0177)	0.0163 (0.015, 0.0178)	0.0162 (0.015, 0.0176)	0.0163 (0.015, 0.0177)	0.0164 (0.0151, 0.0179)	0.0165 (0.0152, 0.018)
Secondary school	0.0161 (0.0131, 0.0189)	0.016 (0.0131, 0.019)	0.0161 (0.0132, 0.0189)	0.0254 (0.0213, 0.0287)	0.0253 (0.0212, 0.0286)	0.0254 (0.0214, 0.0286)	0.0254 (0.0215, 0.0286)	0.0255 (0.0214, 0.0288)	0.0258 (0.0215, 0.0292)
None of above	0.0197 (0.0121, 0.0301)	0.0197 (0.0122, 0.0302)	0.0196 (0.0121, 0.0299)	0.0243 (0.0164, 0.0366)	0.0243 (0.0163, 0.0367)	0.0248 (0.017, 0.0372)	0.025 (0.0175, 0.0374)	0.0243 (0.0163, 0.0367)	0.0244 (0.0162, 0.037)

Ethnicity									
Asian	0.0054 (0.0029, 0.0079)	0.0054 (0.0028, 0.0079)	0.0054 (0.0028, 0.008)	0.0089 (0.0057, 0.0131)	0.0089 (0.0057, 0.0131)	0.0088 (0.0056, 0.0132)	0.0089 (0.0056, 0.0132)	0.009 (0.0057, 0.0132)	0.0089 (0.0056, 0.0132)
Black	0.0143 (0.0108, 0.0177)	0.0144 (0.0109, 0.0178)	0.0141 (0.0107, 0.0175)	0.0222 (0.0179, 0.0261)	0.0223 (0.0179, 0.0261)	0.022 (0.0178, 0.0256)	0.022 (0.0179, 0.0256)	0.0224 (0.018, 0.0263)	0.0227 (0.0183, 0.0267)
Other	0.0077 (0.0043, 0.0111)	0.0077 (0.0042, 0.0112)	0.0077 (0.0042, 0.0111)	0.0114 (0.0076, 0.0151)	0.0114 (0.0077, 0.0151)	0.0114 (0.0076, 0.0151)	0.0117 (0.008, 0.0155)	0.0114 (0.0077, 0.0151)	0.0115 (0.0076, 0.0153)
White	0.0086 (0.008, 0.0092)	0.0086 (0.008, 0.0092)	0.0086 (0.008, 0.0092)	0.0145 (0.0138, 0.0153)	0.0146 (0.0138, 0.0154)	0.0146 (0.0138, 0.0154)	0.0146 (0.0139, 0.0154)	0.0147 (0.0139, 0.0155)	0.0148 (0.014, 0.0156)

Abbreviation: PLCO, Prostate, Lung, Colorectal, and Ovarian (PLCO) Cancer Trial. LLP v2 and v3 refer to the Liverpool Lung Project models versions 2 and 3;
UCLFull-D and UCL-D predict risk of lung cancer death; UCLFull-I and UCL-I predict risk of lung cancer occurrence; LCDRAT, Lung Cancer Death Risk Assessment Tool; LCRAT, Lung Cancer Risk Assessment Tool. Note that the LCDRAT, LCRAT, and PLCOm2012 models were developed in the control arm of the PLCO cohort. The PLCOm2012 was originally developed to predict 6-year risk of developing lung cancer. As Brier scores depend on prevalence, we present results for the PLCOm2012 against 5-year outcomes for the purposes of comparison

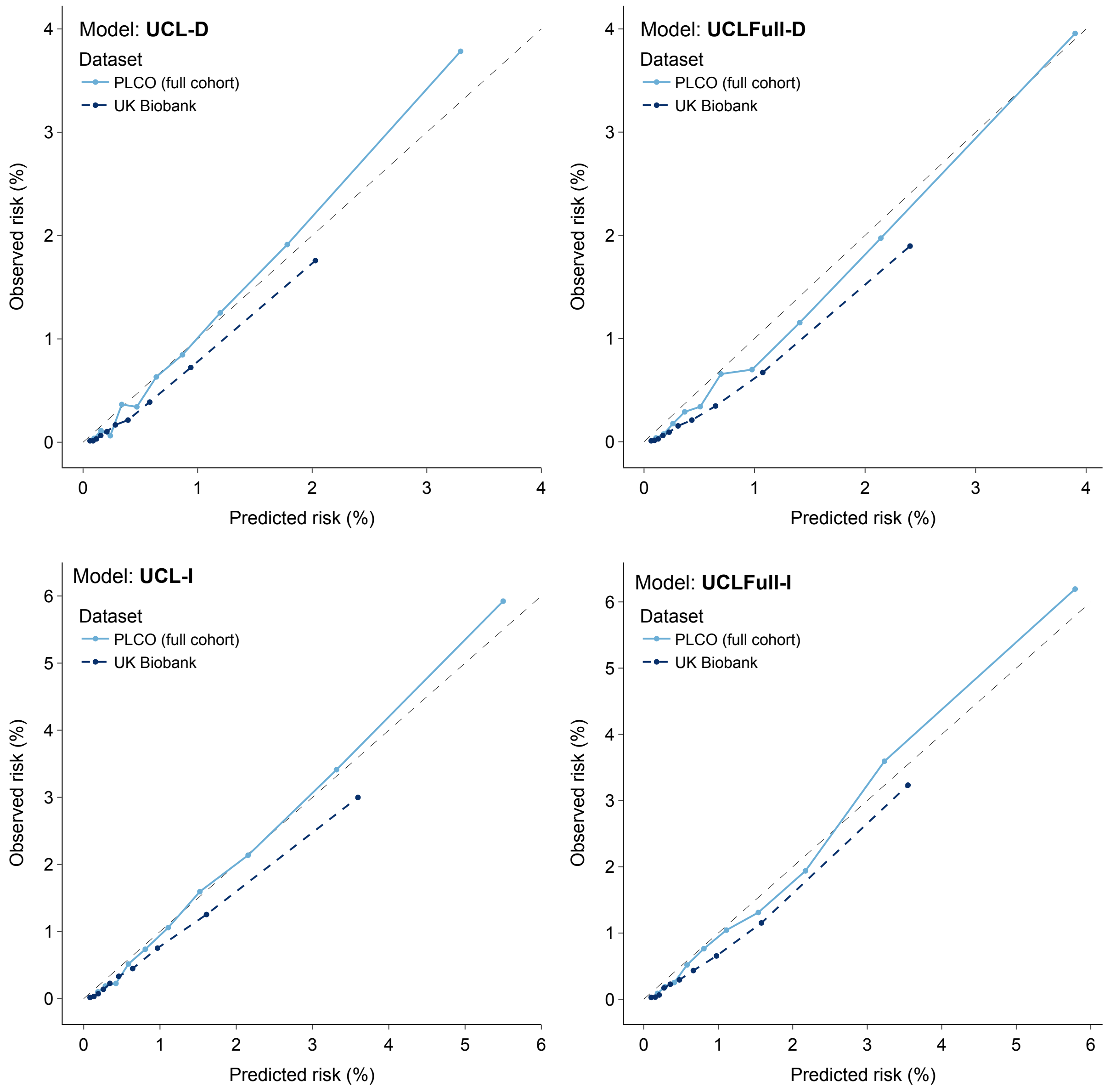

Fig L: Calibration curves for UCL models in the whole PLCO cohort

Calibration curves showing observed against predicted risks in the PLCO full cohort (light blue) and UK Biobank (dark blue). The 45-degree lines in grey indicate perfect calibration.

Abbreviations: PLCO, Prostate, Lung, Colorectal and Ovarian Cancer Screening (PLCO) Trial; UCL-D and UCLFull-D predict lung cancer death; UCL-I and UCLFull-I predict lung cancer occurrence.

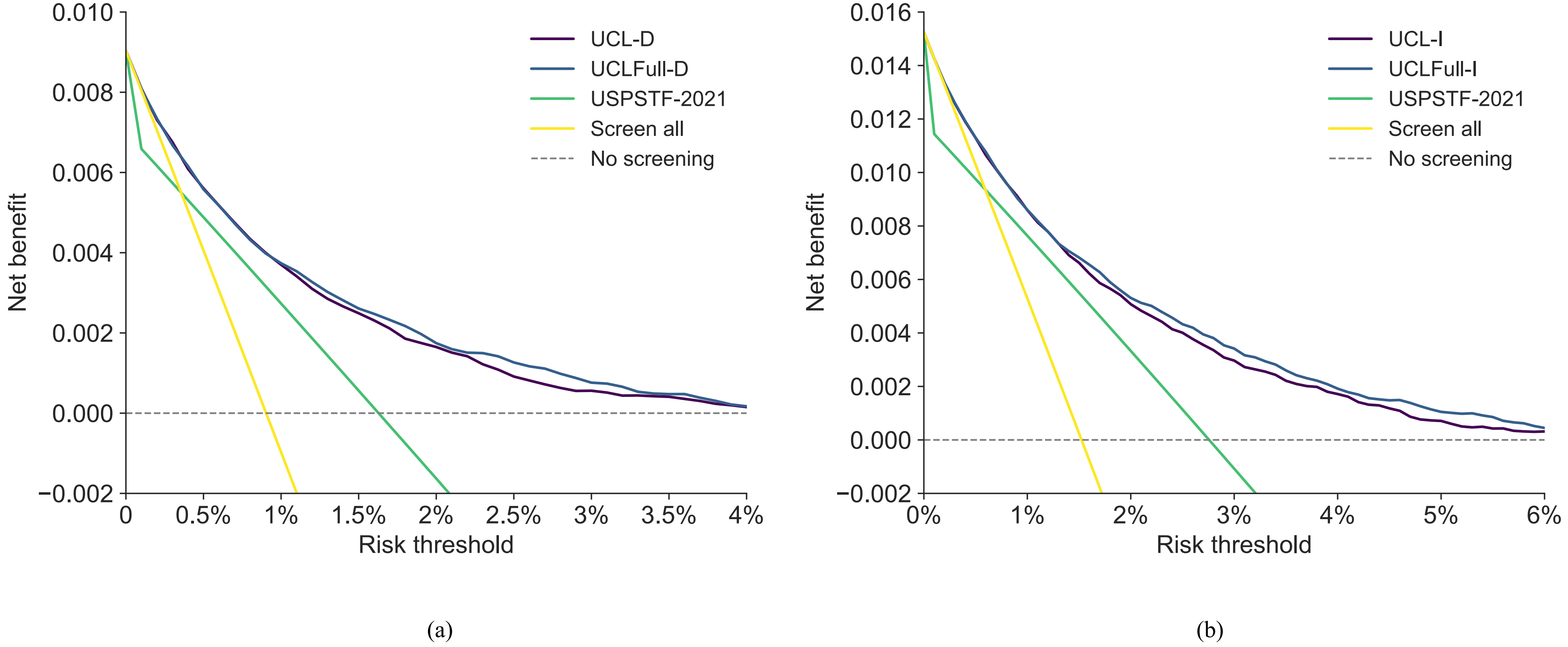

Fig M: Net benefit of UCL models in the whole Prostate, Lung, Colorectal, and Ovarian Cancer Screening (PLCO) Trial cohort

Net benefit of UCL-D and UCL-I across a range of thresholds of models predicting 5-year risk of death from lung cancer (a) and developing lung cancer (b) compared against UCLFull-D, UCLFull-I, and the United States Preventive Services Taskforce (USPSTF)-2021 screening eligibility criteria in the Prostate, Lung, Colorectal, and Ovarian Cancer Screening Trial (PLCO) cohort.

Table R: Sensitivity and sensitivity of UCLFull-D and UCLFull-I at specified risk thresholds in the PLCO dataset

	Risk threshold (%)	Sensitivity (95% CI)	Specificity (95% CI)
Predicting 5-year risk of death from lung cancer			
UCLFull-D	0.74	0.849 (0.821, 0.874)	0.574 (0.571, 0.578)
USPSTF-2021	-	0.775 (0.746, 0.809)	0.574 (0.570, 0.578)
Predicting 5-year risk of developing lung cancer			
UCLFull-I	1.18	0.843 (0.822, 0.863)	0.577 (0.573, 0.580)
USPSTF-2021	-	0.777 (0.758, 0.802)	0.576 (0.572, 0.579)

Abbreviations: CI, confidence intervals; PLCO, Prostate, Lung, Colorectal, and Ovarian Cancer Screening Trial; USPSTF, United States Preventive Services Taskforce. Risk thresholds set using a fixed population approach at a level that would screen an equivalent number as the USPSTF-2021 in the entire PLCO dataset.

References

1. Sperrin M, Martin GP, Sisk R, Peek N. Missing data should be handled differently for prediction than for description or causal explanation. J Clin Epidemiol. 2020;125: 183–187. doi:10.1016/j.jclinepi.2020.03.028

2. Wilson S. Miceforest. [accessed 24 Feb 2022]. Available from: https://github.com/AnotherSamWilson/miceforest

3. White IR, Royston P, Wood AM. Multiple imputation using chained equations: Issues and guidance for practice. Stat Med. 2011;30: 377–399. doi:10.1002/sim.4067

4. Alaa A, van der Schaar M. AutoPrognosis: Automated Clinical Prognostic Modeling via Bayesian Optimization with Structured Kernel Learning. Proceedings of the 35th International Conference on Machine Learning. PMLR. 2018; 139–148. Available from: https://proceedings.mlr.press/v80/alaa18b.html

5. Imrie F, Cebere B, McKinney EF, van der Schaar M. AutoPrognosis 2.0: Democratizing diagnostic and prognostic modeling in healthcare with automated machine learning. PLOS Digit Health. 2023;2: e0000276. doi:10.1371/journal.pdig.0000276

6. Freund Y, Schapire RE. A Decision-Theoretic Generalization of On-Line Learning and an Application to Boosting. J Comput System Sci. 1997;55: 119–139. doi:10.1006/jcss.1997.1504

7. Scikit-learn. An AdaBoost Classifier. [accessed 10 Jan 2023]. Available from: https://scikit-learn.org/stable/modules/generated/sklearn.ensemble.AdaBoostClassifier.html#sklearn.ensemble.AdaBoostClassifier

8. Pedregosa F, Varoquaux G, Gramfort A, Michel V, Thirion B, Grisel O, et al. Scikit-learn: Machine Learning in Python. J Mach Learn Res. 2011;12: 2825–2830.

9. Prokhorenkova L, Gusev G, Vorobev A, Dorogush AV, Gulin A. CatBoost: Unbiased Boosting with Categorical Features. Proceedings of the 32nd International Conference on Neural Information Processing Systems. 2018; 6639–6649. doi:10.5555/3327757.3327770

10. Yandex. CatBoost. [accessed 10 Jan 2023]. Available from: https://catboost.ai/en/docs/

11. Ke G, Meng Q, Finley T, Wang T, Chen W, Ma W, et al. LightGBM: A highly efficient gradient boosting decision tree. Proceedings of the 31st International Conference on Neural Information Processing Systems. 2017; 3149–3157. doi:10.5555/3294996.3295074

12. Microsoft. LightGBM. [accessed 10 Jan 2023]. Available from: https://lightgbm.readthedocs.io/en/v3.3.4/index.html

13. Chen T, Guestrin C. XGBoost: A Scalable Tree Boosting System. Proceedings of the 22nd ACM SIGKDD International Conference on Knowledge Discovery and Data Mining. 2016; 785–794. doi:10.1145/2939672.2939785

14. XGBoost. [accessed 10 Jan 2023]. Available from: https://xgboost.readthedocs.io/en/stable/index.html

15. Akiba T, Sano S, Yanase T, Ohta T, Koyama M. Optuna: A Next-generation

Hyperparameter Optimization Framework. Proceedings of the 25th ACM SIGKDD International Conference on Knowledge Discovery and Data Mining. 2019; 2623–2631. doi:10.1145/3292500.3330701

16. Zhao Y, Wang X, Cheng C, Ding X. Combining Machine Learning Models and Scores using combo library. Thirty-Fourth AAAI Conference on Artificial Intelligence. 2020. doi: 10.1609/aaai.v34i09.7111

17. Toumazis I, Bastani M, Han SS, Plevritis SK. Risk-Based lung cancer screening: A systematic review. Lung Cancer. 2020;147: 154–186. doi:10.1016/j.lungcan.2020.07.007

18. Tammemägi MC, Katki HA, Hocking WG, Church TR, Caporaso N, Kvale PA, et al. Selection criteria for lung-cancer screening. N Engl J Med. 2013;368: 728–736. doi:10.1056/NEJMoa1211776

19. Katki HA, Kovalchik SA, Berg CD, Cheung LC, Chaturvedi AK. Development and Validation of Risk Models to Select Ever-Smokers for CT Lung Cancer Screening. JAMA. 2016;315: 2300–2311. doi:10.1001/jama.2016.6255

20. Field JK, Vulkan D, Davies MPA, Duffy SW, Gabe R. Liverpool Lung Project lung cancer risk stratification model: calibration and prospective validation. Thorax. 2021;76: 161–168. doi:10.1136/thoraxjnl-2020-215158

21. Therneau TM. A Package for Survival Analysis in R. [accessed 18 Jan 2023]. Available from: https://CRAN.R-project.org/package=survival

22. Harrel FE. Regression Modelling Strategies. [accessed 18 Jan 2023]. Available from: https://cran.r-project.org/web/packages/rms/index.html

23. Lundberg SM, Lee S-I. A Unified Approach to Interpreting Model Predictions. Proceedings of the 31st International Conference on Neural Information Processing Systems. 2017; 4768–4777. doi:10.5555/3295222.3295230

24. Lundberg SM, Erion G, Chen H, DeGrave A, Prutkin JM, Nair B, et al. From Local Explanations to Global Understanding with Explainable AI for Trees. Nat Mach Intell. 2020;2: 56–67. doi:10.1038/s42256-019-0138-9

25. Van Calster B, Vickers AJ, Pencina MJ, Baker SG, Timmerman D, Steyerberg EW. Evaluation of markers and risk prediction models: overview of relationships between NRI and decision-analytic measures. Med Decis Making. 2013;33: 490–501. doi:10.1177/0272989X12470757
