# Supplementary material for: Assessing eligibility for lung cancer screening using parsimonious ensemble machine learning models: A development and validation study
Source: PLoS Med. 2023 Oct 3;20(10):e1004287. doi: 10.1371/journal.pmed.1004287 (PMC10547178; doi:10.1371/journal.pmed.1004287)

Assessing eligibility for lung cancer screening: Parsimonious multi-country ensemble machine learning models for lung cancer prediction

The following comments are presented in approximate chronological sequence as they occurred reading through the manuscript and do not reflect their rank importance. Some repetition may occur in comments, please ignore. Some comments made early on may have been explained in the text later on, please ignore.

Model parsimony by limiting the number of predictors may be more than offset by the complexity of the ensemble algorithm and accessing and running it.

“In this study, we hypothesized that using ensemble machine learning with training data spanning different geographic regions, populations, and average risk levels, we could develop predictive models for lung cancer screening with a minimum number of features that has broad applicability.”

“For model development, we first used data on 216,714 ever-smokers without a prior history of lung cancer from the UK Biobank24 before creating a multi-country dataset that combined UK Biobank and US National Lung Screening Trial (NLST)8 data (n=26,616)”
In many instances, pooling effects from different datasets is done with less bias by using meta-analytic methods than just pooling the rows of data together. This probably applies to studies of association more than prediction, but the two often are interconnected.
It is unclear why the model was first developed in the UK Biobank data and then again in the pooled UK Biobank data plus NLST data, and what influence the earlier development had on the later model?

“We selected the NLST because it is geographically distinct, includes a higher risk cohort, and has greater ethnic diversity than the UK Biobank.”
This may be a disadvantage in that the criteria used to select individuals has been shown to miss many individuals who are diagnosed with lung cancer in follow-up. Not including those excluded from the NLST criteria could lead to a model that underperforms in those individuals.

Regarding PLCO sample: “Two external validation cohorts: chest radiography arm & all participants”
These are overlapping and the former is nested in the latter, so it should not leave the impression of totally new validation.

The authors claim to use only 3 predictors, age, smoking duration and pack-years. However, pack-years is not collected directly from individuals. It is constructed from smoking intensity (cigs/day) and smoking duration. That extra step to construct pack-years requires smoking intensity data and an arithmetic step so is tantamount to 4 step data collection.

Figure 1. “(b) shows details of the UCL-D model, including the weights attributed to each pipeline in generating a single prediction for the five-year risk of lung cancer for any individual.”
Logistic and Cox regression models can produce estimates of true absolute risk (probability of new outcome per unit time). However, many machine learning methods do not produce true absolute risks, even though they are made to appear as such by forcing values to lie between 0 and 1.

How are the ensemble model risk scores made to estimate absolute risks? Will these risk differ in different populations sampled? How do we know that the ensemble model score thresholds will not be considerably off in different populations (Note that the model development and testing were in similar populations in this study.)

Figure 1c. Which dataset the figures come from is not described.

Figure 1, “Interestingly, in the subsequent subfigure showing the relationship between pack-years and lung cancer death, we see that there are distinct clusters of individuals based on their smoking duration projecting as a fan. This relationship between smoking duration and pack-years mirrors that seen in the previous sub-figure, with duration trumping quantity of cigarettes smoked unless both are high. In other words, those individuals who smoke for short periods of time have a lower predicted risk, even if they smoke relatively large quantities. This reflects our understanding of lung biology and the ability of the lung to repair itself if an individual stops smoking.52”
Some of the patterns observe between smoking and lung cancer are model driven and data driven more than biologically driven, so “interesting distinct clusters projected as a fan”, may be over interpretation. For example, in the PLCO smoking intensity and thus pack-years was collected in general rounded units, i.e., 10, 20, 30, 40, ….per day.
Similarly, importance of smoking intensity and duration can fluctuate, depending on how they are modeled.

eFigure 3, “80,659 ever smokers included in sensitivity analyses”
Is the “sensitivity” analysis the same as “validation” analysis?
A flow diagram usually divides and separates the population as it goes to different levels. In this case, it does not do so. This initially led to confusion regarding to the purpose and interpretation of the “sensitivity” analysis.

“In sensitivity analyses presented in the Appendix, we report model performance in the full PLCO dataset (n=80,659).”
In what way does this analysis provide a sensitivity analysis?

“We developed models to predict the absolute cumulative risk of two outcomes within five years from baseline:”
It is unclear as to whether a ensemble machine learning “score” is developed versus a true “absolute cumulative risk”? Fore example, a logistic regression model produces estimates of probability of outcome per specified time. Do the machine learning methods give the same thing? Are the model estimates directly poolable?

Were predictors preselected or part of trialing? Where along the modeling process was it decided to use only age, smoking duration and pack-years?

“We trialled model algorithms including logistic regression, random forests and state-of-the art Gradient Boosting approaches (see eMethods, eFigures 7-8, and eTables S2-3).”
The order of eFigures jumps around not in sequence.

Variable Selection
“For pragmatic reasons, we considered candidate predictors from the UK Biobank that were also present in the NLST and PLCO. We settled on our final list of predictors based on the

literature, domain expertise, variable distributions, generalisability to multiple settings, and model discrimination in the UK Biobank."
This does not explain how the final model was pruned to three out of many possible predictors.

"We used optimism-corrected metrics for internal validation."
Optimism-corrected metrics for internal validation usually require going through all steps of the model development process, which includes in this case selection of models, preprocessing, parameter tuning, as well as selection of predictors. Was this done? How?

AUC is a rank order estimator so bad calibration is not detected.

PLCOm2012 and other incidence models also predict death better than incidence. (See ten Haaf et al. 20219)

eTable 2. Hyperparameters
For AdaBoost9–11 it shows a large number of hyperparameters that had to be selected/optimized, which is done in the preprocessing phase of model development:
n_estimators 10
learning_rate 0.1
LightGBM14,15
boosting_type 'gbdt'
learning_rate 0.1
max_depth 6
reg_alpha 9.65x10-6
In Figure 1 (b) and eFigure 8 it describes for AdaBoost that no predictor pre-processing was done.
Part of the problem is that at times different pre-processing is described, that is for predictors and then for models. The full comprehensive pre-processing required should be described to get a better sense of what is required to develop each component model.

Note that death is easier to predict and performance metrics for death are better than for LCA incidence. See eTable 11.

"we also compared against Cox models developed using the same dataset (see eMethods), and the constrained versions of the LCDRAT, LCRAT, and PLCOm2012 models."
The Cox model was developed and parameterized in the same dataset, but the other models were not and were merely "constrained". This may limit the comparisons.

Results
The UCL-I had an AUC of 0.787 (95% CI: 0.771-0.802), an E/O ratio of 1.0 (0.92-1.07), and a Brier score of 0.0153 (0.0142-0.0164).
This is essentially a tie with existing best models (PLCOm2012 and LCRAT) for lung cancer incidence.

"UCL models showed good calibration in external validation in the PLCO intervention arm, particularly at predicted risk between 1-2% at which risk thresholds are commonly set. At these thresholds there was modest underprediction with the LCDRAT, LCRAT, and PLCOm2012 models in the PLCO intervention arm."

Different models have different calibration depending on the base population the models are derived from, so one would not expect direct comparisons of thresholds across models.
It is still unclear as to how true absolute risk is derived from the ensemble models?
Overall Performance (Appendix Table S11)
"Nevertheless, the same pattern was observed: UCL-I had a Brier score of 0.0153 (95% CI: 0.0142-0.0164), LCRAT a score of 0.0152 (95% CI: 0.0143-0.0164), and LLP version 2 a score of 0.0153 (95% CI: 0.0143-0.0165)."
eTable 11 shows that the Brier score for UCL-I, LCRAT and PLCOm2012 are all 0.006 and for LLPv2 is 0.0062 (higher is worse). The authors statement as worded seems incomplete and inaccurate.

Figure 3. Does the consistent higher Net Benefit for the good incidence models (B) over the death models (A) suggest that applying incidence models is preferred to death models? I realize that which type of model to use is more complicated that this.

Discussion
"Using three unambiguous variables but with equivalent or improved performance, the UCL models could be completed more easily online or in primary healthcare, simplifying the implementation of lung cancer screening"
This conclusion depends on how simple and accessible the program is online. For example, the PLCOm2012 risk prediction model has several versions, which are available in smart phone apps and are built into hospital medical records systems such as EPIC, and spreadsheet calculators are available that work readily on computers and laptops offline.

"In keeping with Katki and colleagues,19 we found that UCL-D, predicting the risk of death from lung cancer, had greater discrimination than models predicting lung cancer occurrence."
But this is true generally for incidence models. They as well predict lung cancer death better than they do incidence – with higher AUCs. See ten Haaf 2019. The problem with death models is the they generally have had poor calibration. In the PLCO data, over ½ of the lung cancer deaths in those who were diagnosed with lung cancer in five years of follow-up from baseline died after the five years period. They would be excluded from a death model.

"Given this, UCL-D would be the more appropriate model to consider for implementation."
The problem with death models such as the LCDRAT, is that they have generally been calibrated poorly. See previous comment.

The market is currently being flooded with new attempts at models and many of them use machine learning approaches. None have established themselves as being substantially better than existing validated models, some of which are being used in practice.

"Code and model availability
To facilitate use of the UCL models, we have developed a website and have made the models themselves available (https://github.com/callta/lung_cancer_risk_models) as a package. The underlying code for AutoPrognosis is available from https://github.com/vanderschaarlab/AutoPrognosis."

The ensemble model presented in the current manuscript provides predictive performance that is possibly similar to the LCDRAT, LCRAT or PLCOm2012. The primary claim to superiority is that it is simpler and easier to use because it has fewer predictor variables.

However, the assertion that collection of data for full risk prediction models is difficult has been exaggerated and researchers using or evaluating the PLCOm2012 model in the UK and Europe state that it is easy and fast and can be completed in 4 minutes. In the Ontario Lung Cancer Screening Pilot/Program focus groups, post-risk assessment participant satisfaction surveys and stake holder risk assessment administrator interviews describe high satisfaction and no important issues.
The issue concerning the ensemble model hinges on is how easily is it to access and implement. It seems not to be easily transportable and useable independent of internet access to a website. This reviewer attempted several times to gain access to the models to attempt implementation and testing but got error message displayed below. This left the reviewer with a sense of uncertainty regarding an important aspect of model functionality.

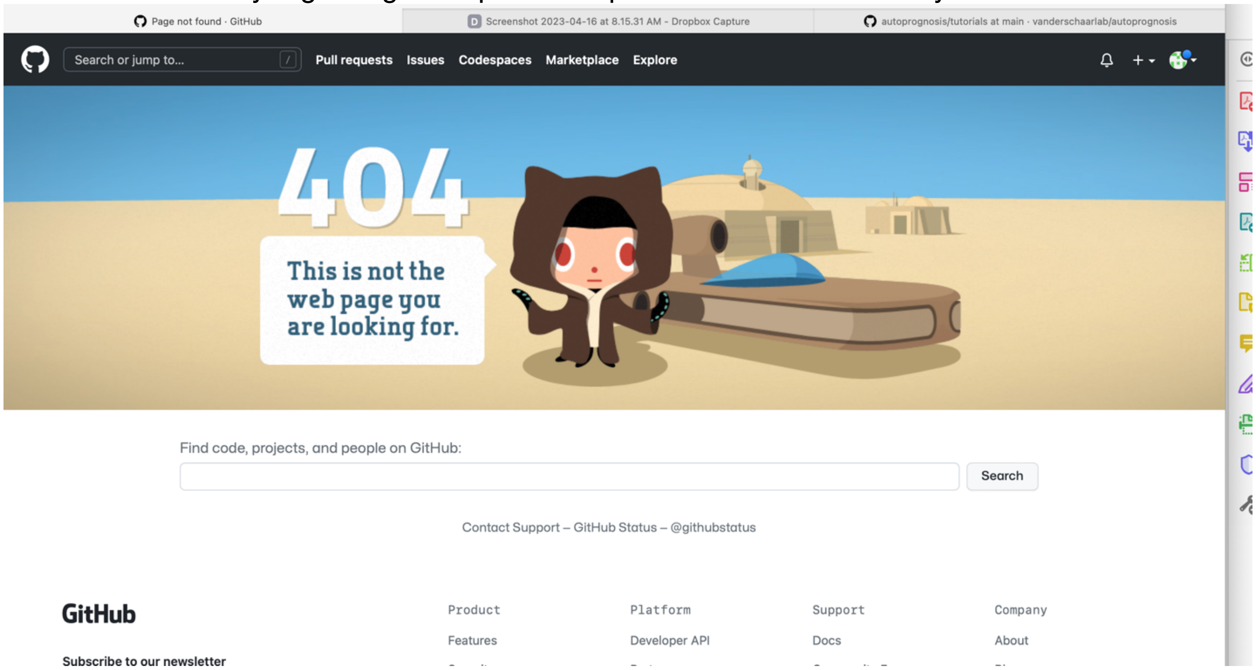

Supplement: Attachment — Submitted filename: PLOSmed_REV_NOTES_11APR2023.pdf [file pmed.1004287.s002.pdf]
