# Supplementary material for: Assessing eligibility for lung cancer screening using parsimonious ensemble machine learning models: A development and validation study
Source: PLoS Med. 2023 Oct 3;20(10):e1004287. doi: 10.1371/journal.pmed.1004287 (PMC10547178; doi:10.1371/journal.pmed.1004287)
Supplement: Attachment — Submitted filename: response letter.pdf [file pmed.1004287.s004.pdf]

Dear Dr Dodd,

Thank you for the opportunity to further revise the manuscript and for the feedback and comments.

Requests from the editors:

GENERAL

Thank you for your detailed and considered responses to previous editor and reviewer comments. Please see below for further comments which we require that you address in full.

Please cite your Supporting Information as outlined here: https://eur01.safelinks.protection.outlook.com/?url=https%3A%2F%2Fjournals.plos.org%2Fplosmedicine%2Fs%2Fsupporting-information&data=05%7C01%7Ct.callender%40ucl.ac.uk%7C781bdd89eab2448fdd9008db7e42d09b%7C1faf88fea9984c5b93c9210a11d9a5c2%7C0%7C0%7C638242597654582928%7CUnknown%7CTWFpbGZsb3d8eyJWIjoiMC4wLjAwMDAiLCJQIjoiV2luMzIiLCJBTiI6Ik1haWwiLCJXVCI6Mn0%3D%7C3000%7C%7C%7C&sdata=E9ePkj9b3P3TcGTQgmpSkukyUismiV6rGElOn%2BUqevk%3D&reserved=0

Done. We have renamed figures and tables in the Appendix to Fig Sx and Table Sx, accordingly.

* Considering that the comments from the statistical and subject reviewer (please see below) pertain to further clarifications of your model and analyses, and in view of the fact that revisions have the potential to alter the data, it is for this reason that we have invited a further major revision. *

DATA AVAILABILITY STATEMENT

The Data Availability Statement (DAS) requires revision. For each data source used in your study:
a) If the data are freely or publicly available, note this and state the location of the data: within the paper, in Supporting Information files, or in a public repository (include the DOI or accession number).
b) If the data are owned by a third party but freely available upon request, please note this and state the owner of the data set and contact information for data requests (web or email address). Note that a study author cannot be the contact person for the data.
c) If the data are not freely available, please describe briefly the ethical, legal, or contractual restriction that prevents you from sharing it. Please also include an appropriate contact (web or email address) for inquiries (again, this cannot be a study author).

Done. Now reads:

> UK Biobank, NLST, and PLCO data were used on license (references 68073, NLST-806 and PLCO-801, respectively).
>
> These data are subject to material transfer agreements and cannot be shared directly. However, researchers can apply for these data from the UK Biobank (https://www.ukbiobank.ac.uk/) and the US National Institutes of Health (PLCO: https://cdas.cancer.gov/plco/; NLST: https://cdas.cancer.gov/nlst/).

TITLE

The title is quite long, please revise. Your title must be nondeclarative and not a question. It should begin with main concept if possible. "Effect of" should be used only if causality can be inferred, i.e., for an RCT. Please place the study design ("A randomized controlled trial," "A retrospective study," "A modelling study," etc.) in the subtitle (ie, after a colon).
Suggest - 'Assessing eligibility for lung cancer screening using parsimonious multi-country ensemble models: A machine-learning study' or similar.

We have revised the title to: "Assessing eligibility for lung cancer screening using parsimonious ensemble machine learning models: A development and validation study"

AUTHOR SUMMARY

Line 67 – suggest, 'Combining predictive performance with administrative simplicity could allow for streamlined delivery of risk-tailored lung cancer screening.' This statement is a little vague suggest elaborating. How does the simplified approach streamline things – reaches regions with limited availability of patient data, for example.
Line 69 – suggest, 'Further research should focus on the application of this model design to other conditions such as…'
In the final bullet point of 'What Do These Findings Mean?', please describe the main limitations of the study in non-technical language.

Done. We have re-written the and expanded the 'What do these findings mean?' section in accordance with your points as follows:

- Relatively few predictors are necessary to determine lung cancer risk amongst those who have ever-smoked cigarettes.
- Future research should focus on the application of our modelling approach to other conditions such as cardiovascular disease, diabetes, and chronic kidney disease.
- This study has key limitations as it is based on past data from the US and UK, so prospective evaluation in different countries and regions should be considered.

INTRODUCTION

Line 110-111 – suggest 'per year' Is the obstacle you refer to cost? Please clarify

Done. We have changed a whole year to a year and adjusted the last lines to clarify such that the paragraph now reads:

> Furthermore, the risk models currently in use are a challenge to implement. In the UK, eligibility for lung cancer screening pilots is based on the PLCOm2012 and Liverpool Lung Project risk models, requiring 17 unique variables, few of which are routinely available [23]. Collecting these variables from an individual who is potentially eligible and explaining the results currently averages between five and ten minutes. To determine the screening eligibility of one million people would therefore require between 48 and 95 full-time staff a year. In the UK, there are an estimated 6.8 million ever-smokers aged 55-74 who are potentially eligible for lung cancer screening, with another 500,000 turning 55 on average each year [24]. As lung cancer screening is just one of several risk-based programmes that are either in development or in use, in their current form, these assessments present a formidable obstacle to the effective implementation of national screening programmes.

Line 148 – '(PLCO) [28] trial' please add a space between the sets of brackets

Done

METHODS and RESULTS
Line 185 – '(SHAP) [33]' please add a space between the sets of brackets

Done

Line 189 – please indicate where in the appendix that the details you refer to can be found

We have changed this sentence so that it now reads:

> Further details are available in sub-section Variable Importance and Interaction in S1 Appendix.

DISCUSSION
Thank you for structuring your discussion as suggested. Please expand on the limitations of your study which are rather brief.

Done. We have added several sentences to the limitations such that this paragraph now reads:

> This study has several limitations. We have used retrospective data, such that findings may differ if used to prospectively determine screening eligibility. However, both the PLCOm2012 and the LLP models have been studied in prospective settings, establishing the benefits of risk-model against risk-factor-based screening. By benchmarking against these models, we can be confident in the performance of our models in a screening programme. To confirm the generalisability of our models, validation in datasets from beyond the US and UK will be the subject of further work. Our analyses have been performed in research cohorts rather than routinely collected electronic health records that may better reflect the broader population. In keeping with the models used as benchmark comparators in this work, the UCL models may not perform to the same extent in routinely collected electronic health records as smoking data are not usually present in the same depth [54]. Nevertheless, screening programmes are unlikely to rely on existing electronic health records given known challenges of missing and inaccurately coded predictors [55]. Finally, our risk models exclude never-smokers. To date, no risk model has been able to discriminate those never smokers with sufficient risk to meet existing criteria for lung cancer screening.

We could not find a section which details the strengths of your study, please include.

Done. We have added the following paragraph prior to the limitations paragraph in the discussion:

> This study has strengths. We used large prospective cohorts for model development and validation. Our external validation cohort is both temporally and geographically distinct. We used robust methods for model development and internal validation whilst externally validating our models extensively using multiple approaches and in a wide range of sub-groups. Further, we benchmarked our models against leading comparators. Moreover, by using few, unambiguous, variables, our models could be widely applied after further validation and, where necessary, recalibration. Finally, we have made our models openly available for independent assessment.

Lines 366 – 374 – suggest moving this text to the second paragraph it seems out of place here (and the opening sentence a bit repetitive this far through the discussion. Please also remove the phrase ‘state-of the-art’

Done, we have moved this text so that they now make up the second paragraph of the discussion. We have also removed state-of-the-art – apologies for having overlooked this on the last round of revisions.

Line 386 – please remove ‘In summary’

Done, we have removed and adjusted to ‘in conclusion’. Please let us know if you’d like the sentence instead as is without in conclusion.

> In conclusion, we used ensemble machine learning to explicitly maximise model parsimony, an approach that holds promise in multiple disease areas.

REFERENCES
Please ensure that all web references listed include an access date, ref #7 and #31-34, for example
Is ref #29 a complete reference?

Done.

SUPPORTING INFORMATION
eFig7 – please define SHAP in the caption/footnote
Please ensure that the required referencing format is applied to the supporting files.
Please list up to but no more than 6 author names followed by et al.
Please ensure that journal name abbreviations are those found in the National Center for Biotechnology Information (NCBI) databases.
Please ensure that all web references include an access date
Please see our website for other reference guidelines https://eur01.safelinks.protection.outlook.com/?url=https%3A%2F%2Fjournals.plos.org%2Fplosmedicine%2Fs%2Fsubmission-guidelines%23loc-references&data=05%7C01%7Ct.callender%40ucl.ac.uk%7C781bdd89eab2448fdd9008db7e42d09b%7C1faf88fea9984c5b93c9210a11d9a5c2%7C0%7C0%7C638242597654739121%7CUnknown%7CTWFpbGZsb3d8eyJWIjoiMC4wLjAwMDAiLCJQIjoiV2luMzIiLCJBTiI6Ik1haWwiLCJXVCI6Mn0%3D%7C3000%7C%7C%7C&sdata=AuHmw9bIYBo%2Fs1U1ANcyhOdy%2FAAHv3FXgLKHtaYUOEA%3D&reserved=0

Done.

SOCIAL MEDIA
If not already done so, to help us extend the reach of your research, please detail any Twitter handles you wish to be included when we tweet this paper (including your own, your coauthors’, your institution, funder, or lab) in the manuscript submission form when you re-submit the manuscript.

Comments from the reviewers:

Reviewer #1: We thank the authors for substantively addressing our previous comments, and the emphasis on recalibration for future application in the responses to other reviews. A couple of points

might yet be considered:

1. In the response to the original Point 5, it is clarified that the 216,714 UK Biobank and 26,616 US NLST subjects were combined without any weighting. However, the additional value of including the US NLST data is then not immediately clear, as it might be dominated by the UK Biobank data.

It might thus be considered to perform brief ablation experiments with only Biobank/only NLST data, to quantify the added value of including additional data sources.

As you suggest, we did separately train several models in the UK Biobank and the UK Biobank + NLST (combined dataset) to understand if there was value to this approach. The key difference was that our full model for predicting lung cancer within 5 years developed in the UK Biobank was poorly calibrated relative to the model developed in the combined datasets, despite having a similar AUC. This same finding held in the parsimonious three variable version. For congruence and clarity throughout we decided to keep with the combined datasets.

Overall, our rationale for including the NLST subjects was to diversify the data on which the models were trained as the NLST includes a substantially higher risk cohort with more ethnic diversity than the UK Biobank (12% non-white vs ~3.5% non-white). Further, by including the NLST data, we increased the number of outcomes for model training by nearly 50% (an additional 719 lung cancers and 365 deaths), hence why we felt that no form of weighting was required. Also, in the NLST there were twice as many outcomes in non-white ethnicities than the UK Biobank (28 vs 13 deaths from lung cancer within five-years). Although small, as there are noted ethnic difference in lung cancer risk, we felt that any additional cases would be worth keeping. The numbers of individuals from non-white ethnicities remains small in both our development and validation sets. Consequently, to be confident of any added benefit (or lack thereof) with these kind of combined / augmented data methodological approaches would require multiple further diverse datasets to be able to tease out whether there are true generalisability (+- performance) improvements. This will be the subject of further work.

As we are keen to ensure that the main message of the paper – relating to parsimonious models developed with ensembling techniques – is clear, we have not made statements that combining the datasets has improved model performance. Nevertheless, from a practical perspective, as the development cohorts are sensible and the models have been thoroughly validated, it should not detract from the use of our models.

2. Moreover, for the NLST data, the background does not appear to be analyzed as thoroughly as for Biobank data (as in the "Variable recoding, missing data, and multiple imputation" section of the supplementary material). In particular, it is not immediately clear as to the number of subjects progressing to/dying from lung cancer, as clearly laid out for Biobank data in eTable1. It might thus be considered to present the NLST data in more detail too.

We did go into the same detail of analysis. However, the NLST data had overall missingness of <1% and no missing age or relevant smoking data (smoking duration, smoking intensity, and pack-years) (Table 1 in the main manuscript), such that there would be no relevant patterns of missing data amongst smoking variables as found in the UK Biobank for us to present. We have revised and clarified the relevant paragraph in the appendix such that it now reads:

> Overall missingness was <5% for all relevant variables in both the NLST and PLCO datasets. Given the low level of missingness, we generated five imputed NLST and PLCO datasets. In

both cases we used multiple imputation by chained equations with predictive mean matching, implemented as described above. Note there was no missingness amongst age, smoking intensity, and smoking duration variables in the NLST trial.

In terms of the number of NLST participants progressing to / dying from lung cancer, we present these by year of follow-up in Table S8, alongside relevant figures for the UK Biobank and PLCO trial.

Reviewer #2: none

Reviewer #3: All in all, the authors responded well to the comments made by reviewers. I only have minor comments left.

Abstract
"Ensemble machine learning could support the development of highly parsimonious prediction models that maintain the performance of more complex models whilst maximising simplicity and generalizability"
Regarding "maintain the performance of more complex models whilst maximising simplicity and generalizability",
it is difficult to conceptually appreciate that a model that looks at only a limited number of risk factors can be as robust at estimating personal risk as a model that has a wide diverse collection of predictors, which taken together as a package summarize a person's global risk more comprehensively than focussing on a narrow list that consists of three of predictors age and smoking.
The eFig 7 shows the relative importance contribution of predictors in the full model. It shows that combined BMI, quit-years, smoking intensity, family history of lung cancer and COPD, contribute substantially more to prediction than do pack-years or age. Thus, they may be meaningful in some setting.
The model development and validation came from high quality and complete data. When this model is applied to electronic medical record data in which pack-years and duration smoked is incomplete and out of date, then ancillary data from other variables can be valuable in augmenting age/smoking predictors, for example, BMI, history of COPD, family history of lung cancer, personal history of cancer, race, are predictive variable which often at least in part are available in patients EMR which can offset absence or measurement error in smoking data. Thus, it is expected that a fuller model will be more robust than a parsimonious bare-bones model.
The authors describe and explain that their use of model would involve collection of accurate data, not reliance on electronic medical records, which makes my comments regarding EMR less relevant in their setting.

Conceptually, it is likely that there will be some individuals where the collection of additional variables would change predictions slightly, though perhaps not change whether they would receive screening – the ultimate goal of the model. However, we have shown in extensive sub-group analyses that, in the case of ever-smokers where smoking and age have such an important relative impact on predictions, the addition of more variables is of limited, if any, benefit. Fundamentally, a key trade-off that we are arguing is that, when one has presented a model that is performing as well with so few variables, what are the added benefits for a screening programme in searching for additional variables when they are not necessarily coded well and can be time-consuming to collect.

In future work, we will consider ways of considering if different models are required in those for whom predictions are both uncertain and where these uncertainties matter to whether an individual will thus be screened or not (i.e., change whether they are over relevant screening thresholds). This would allow us to maximise parsimony – and thus implementability – for most, whilst only deploying models with more predictors for those who would benefit from this.

Further, in terms of automation in an electronic health record, perhaps additional variables can support prediction models in those cases where pack-years / smoking duration are not either present or are inaccurate. Nevertheless, our analyses have shown that it is still likely that fuller models for use in EHRs will still be primarily driven in this context by age and smoking status / intensity, both of which are present for example in UK primary care records.

However, from a policy perspective, it is unlikely we will be using automated risk prediction from electronic records, no matter how appealing it appears, because of inherent biases and errors. For example, an analysis of over 250 primary care practices and 100,000 individuals in north London recently showed that – despite all being flagged as current smokers – 25% were not or had never been current smokers, whilst there were also differences in coding quality by ethnicity and income deprivation (Dickson et al., 2022a). Within the Clinical Research Practice Datalink of research-grade UK primary care records, amongst ever-smokers, only 10% had a recorded age at which they started smoking (O'Dowd et al. 2021). When it comes to variables that are more subjective, the congruence between education categories when assessed over the phone relative to in-person for UK lung cancer pilots in north London was only 50% (Dickson et al., 2022b). All this suggests that our approach is likely to be of more clinical usefulness for the effective implementation of lung cancer screening than attempting to use EHRs.

We have added the following sentences to the paragraph on limitations in the discussion to discuss this further:

> Our analyses have been performed in research cohorts rather than routinely collected electronic health records that may better the broader population. In keeping with the models used as benchmark comparators in this work, the UCL models may not perform to the same extent in routinely collected electronic health records as smoking data are not usually present in the same depth [54].

Refs:
Dickson et al. 2022a: doi:10.1016/j.lungcan.2022.09.009
Dickson et al. 2022b: doi:10.1136/thoraxjnl-2021-218634
O'Dowd et al. 2021: doi:10.1136/thoraxjnl-2021-217142

"We externally. validated our models amongst 49,593 participants in the chest radiography arm and all
80,659 ever-smoking participants in the US Prostate, Lung, Colorectal and Ovarian Screening Trial (PLCO)."
In the Abstract, the thinking behind this strategy is unclear for readers.

Thank you - we have adjusted the abstract by adding the following sentence to clarify:

> We primarily validated in the PLCO chest radiography arm such that we could benchmark against comparator models developed within the PLCO control arm.

"our prognostic models would be trained on a wider range of participants, with the potentially

improved model performance"
Although the term "prognostic model" has been widely and loosely used in recent years, I prefer to use "prognostic" to describe survival in individuals with cancer (individuals with disease) and not for prediction models describing risk of developing lung cancer in individuals without disease who will in the future develop disease.

Done. We have changed any reference to prognostic models to prediction models.

From response to reviewer #3.
"As the difference in discrimination between the full models and the three-variable models was not statistically significant, we took forward the three-variable models."
Was a p-value or confidence intervals for the difference presented?

With the previous round of reviews, the text was simplified such that this sentence is no longer in the main manuscript. However, the P values were p=0.12 for difference in AUC between UCLFull and UCL-I and p=0.55 for the difference between UCLFull-D and UCL-D in the UK Biobank internal validation. We will make peer review open as is an option with PLOS Medicine should our paper be published, such that readers can see this here.

eTable 14, Overall calibration lower confidence interval for LCRAT is missing second decimal place. Missing second decimal occurs elsewhere too.

Thank you – now corrected.

The eTable 14 indicates that the UCL-D and UCL-I are not well calibrated, with 47% and 35% excess risk estimates in the UCL-D and UCL-I models respectively. That the final pooled estimated E/R is much closer to 1 indicates that the calibration in the non-UK Biobank data may be poorly calibrated in the opposite direction, so that they cancel out.

In the UK Biobank, as noted in eTable 14 (now Table S14), both UCL-D and UCL-I are indeed overpredicting risk overall, in keeping with the LCDRAT and LCRAT models. We decided not to specifically re-calibrate the models for the UK Biobank as this cohort suffers from healthy volunteer bias and the aim is for models that might be suitable for a national population which will include areas with higher risk and areas that have lower risk. As the PLCO cohorts have incident cancer risks that are approximately mid-way between that of the UK Biobank and the high-risk NLST cohort, we expect that this is more likely to reflect the broader population in which our models might be used.

In a few places where we could readily check the results, the numbers did not match. In particular, the AUC and E/O for PLCOm2012 in the PLCO intervention arm and entire sample were underestimated. Do the authors think that this was due to computing issues, program used to prepare estimates or modifications of dataset? Examples are provided:

The differences will be because we used bootstrapping in the PLCO cohorts for our results (central estimates and confidence intervals for AUC / brier / calibration) and due to slight variation in the datasets with imputation (for example there were 74,218 complete samples in the below print-out compared with ~80,000 participants in the full cohort). Results taken from multiple bootstrap resamples would be expected to be more conservative than an AUC on the original dataset. As we used the same bootstrapping approach when comparing all models in the PLCO cohorts, the relative impacts when comparing models will not be affected.

Table 4: Calibration overall and by subgroup in the PLCO chest radiography cohort

Tabel 4 reports Ratio of expected-to-observed cancers with 95% confidence intervals overall for PLCOm2012 to be "0.93 (0.86, 0.99)"
Our STATA results in the PLCO intervention arm are different, with E/O = 0.95 and AUC 0.797.
STATA result:

```
/* . brier lcancer6yr PxbMFP if H_rndgroup==1
Mean probability of outcome   0.0181
                    of forecast    0.0172
display 0.0172 / 0.0181  //   .95027624 E/0 = 0.95
ROC area                    0.7967  p = 0.0000      */
```

eTable 16: Discriminative accuracy (AUC) in the whole PLCO cohort
eTable 16 reports that in the PLCO overall for PLCOm2012 the AUC (95% CIs) are "0.79 (0.78, 0.8)".
When we calculate them with original data and model, the results are 0.80 (.79 to 0.81).
STATA printout

```
/* . roctab lcancer6yr PxbMFP if H_rndgroup<2
                  ROC                  Asymptotic normal
         Obs      area     Std. err.      [95% conf. interval]
     ------------------------------------------------------------
       74,218     0.7995       0.0056        0.78859     0.81038
REPORTED IN PLOSmed manuscript  0.79 (0.78, 0.8)
OUR STATA RESULTS ARE               0.80 (.79 TO 0.81)         */
```
